# Association between triglyceride-glucose indices and incident glaucoma: integrated UK Biobank and Mendelian randomization study

**DOI:** 10.1186/s40662-026-00516-x

**Published:** 2026-10-10

**Authors:** Jianming Xu, Yuelan Gao, Jun Yu, Zihe Xu, Yuzhou Zhang, Chi Pui Pang, Clement C. Tham, Jason C. Yam, Li Jia Chen

**Affiliations:** 1 Department of Ophthalmology and Visual Sciences, The Chinese University of Hong Kong, 4/F Hong Kong Eye Hospital, 147 K Argyle Street, Kowloon, Hong Kong, Hong Kong SAR China; 2 Joint Shantou International Eye Center of Shantou University and the Chinese University of Hong Kong, Shantou, China; 3 Department of Ophthalmology and Visual Sciences, Prince of Wales Hospital, Hong Kong, China; 4 Hong Kong Eye Hospital, Hong Kong, China

**Keywords:** Glaucoma, TyG index, TyG related indices, Biomarkers, UK Biobank

## Abstract

**Background:**

To evaluate the associations of the triglyceride-glucose (TyG) index and related adiposity-integrated indices with incident glaucoma and explore potential causal evidence using Mendelian randomization (MR).

**Methods:**

This prospective cohort study included 396,213 participants without glaucoma of European ancestry from the UK. The TyG index and four related indices, including TyG-body mass index (TyG-BMI), TyG-waist circumference (TyG-WC), TyG-waist-to-height ratio (TyG-WHtR), and TyG-waist-to-hip ratio (TyG-WHR), were assessed. Multivariate Cox models and restricted cubic splines were used to examine the association with incident glaucoma. Associations between the corneal-compensated intraocular pressure (IOPcc) and retinal structures were assessed using regression analyses. The predictive performance was evaluated using extreme gradient boosting (XGBoost). Two-sample MR was conducted to assess the genetic association between the TyG index and glaucoma.

**Results:**

During a mean follow-up of 8.06 years, 6957 cases of incident glaucoma occurred. In fully adjusted models, higher TyG and related indices were associated with increased glaucoma risk, including TyG (hazard ratio [HR] = 1.05, 95% confidence interval [CI]: 1.02–1.08), TyG-BMI (HR = 1.20, 95% CI: 1.14–1.27), TyG-WC (HR = 1.30, 95% CI: 1.23–1.37), TyG-WHtR (HR = 1.26, 95% CI: 1.20–1.31), and TyG-WHR (HR = 1.16, 95% CI: 1.12–1.21). The TyG showed a significant non-linear U-shaped association with incident glaucoma. TyG-related indices were positively associated with IOPcc, although the effect sizes were small and inversely associated with several measurements of retinal layer thickness. The XGBoost model showed good discrimination, with TyG-WHtR contributing the strongest among the TyG-related indices (C-index = 0.851, area under the curve = 0.823). MR provided suggestive evidence for an association between genetically predicted TyG and glaucoma risk (odds ratio = 1.27, 95% CI: 1.00–1.62, *P* = 0.046), although sensitivity estimates were not statistically significant.

**Conclusions:**

TyG-related indices, particularly adiposity-integrated measures, were associated with a higher glaucoma risk. Systemic glucose-lipid metabolic dysfunction and central adiposity may contribute to glaucoma susceptibility. Thus, TyG-related indices may serve as low-cost adjunct indicators for glaucoma risk stratification and external validation.

**Supplementary Information:**

The online version contains supplementary material available at https://doi.org/10.1186/s40662-026-00516-x.

## Background

Glaucoma, a leading cause of irreversible vision loss worldwide, will affect 111.8 million individuals by 2040 [1]. The disease is characterized by progressive apoptosis of retinal ganglion cells (RGCs), which results in thinning of the retinal nerve fiber layer and corresponding visual field defects. Elevated intraocular pressure (IOP) remains the primary, and currently the only modifiable, risk factor for glaucoma [2]. Although current clinical approaches focus predominantly on lowering IOP through medication, laser therapy, and/or surgery, this strategy alone remains inadequate to restore vision [3, 4]. Therefore, the identification of biomarkers for early diagnosis and therapeutic targeting for prevention is crucial for glaucoma management [5].

Diabetes and hyperlipidemia are risk factors for glaucoma, and blood glucose and lipid levels are associated with increased IOP [6, 7]. Recent metabolomic studies have substantiated a significant link between disorders of glucose and lipid metabolism and glaucoma risk [8–10]. In addition, genome-wide association studies (GWASs) have identified diabetes- and lipid-related genes that are associated with glaucoma [11, 12]. By studying glycolipid metabolism in isolation, previous research has overlooked the critical synergy of its components, likely diminishing their individual value as biomarkers for glaucoma.

The triglyceride-glucose (TyG) index was first reported in 2010. The study proposed the formula TyG index =$ln [triglycerides\,(mg/dL) \times glucose\,(mg/dL)/2]$ and demonstrated its high sensitivity and specificity compared to the gold standard euglycemic hyperinsulinemic clamp test. Emerging evidence has shown that the TyG index is a reliable surrogate marker for insulin resistance. It is increasingly used in clinical settings to assess metabolic and vascular diseases, such as diabetes and cardiovascular disease (CVD), and has demonstrated strong diagnostic and predictive capabilities in these areas [13–15]. By contrast, studies exploring the association between the TyG index and ocular diseases remain scarce. Given the contributions of abnormal retinal blood flow and metabolic dysregulation to glaucoma pathogenesis [9, 16], we hypothesized that the TyG index, a marker of insulin resistance, is a relevant and modifiable risk factor for glaucoma. A recent cross-sectional study among participants with hypertension provided initial insights into this relationship [17]. However, its broader applicability may be limited by the study’s cross-sectional design, sample size, and potential for residual confounding, leaving the question of the utility of the TyG index as a clinical biomarker for glaucoma open for further investigation.

**Fig. 1:**
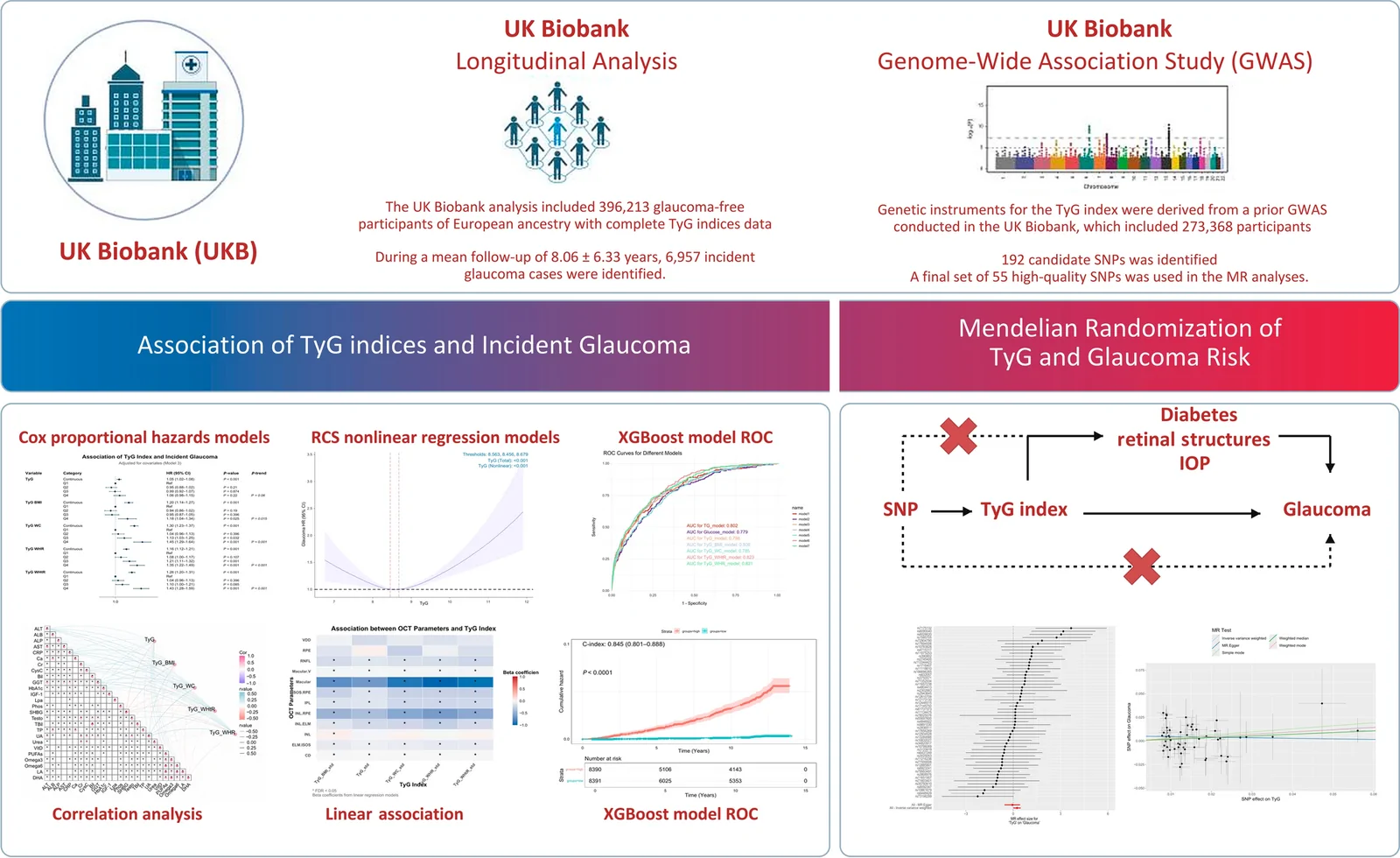
Study design and analytical overview for investigating the association between the triglyceride-glucose (TyG) index and glaucoma in the UK Biobank. The study consists of two main parts: (Left) A longitudinal analysis of 396,213 participants from the UK Biobank, which employed Cox models, restricted cubic splines (RCS), and an XGBoost algorithm to assess the association of TyG-related indices with incident glaucoma, intraocular pressure (IOP), and retinal structures. (Right) A Mendelian randomization (MR) analysis using genetic instruments from a TyG index genome-wide association study (GWAS) to evaluate its potential causal effect on glaucoma risk, with mediation analyses exploring the role of diabetes and retinal features. ROC, receiver operating characteristic; SNPs, single-nucleotide polymorphisms

In this study, we utilized a large-scale, prospective UK Biobank cohort to comprehensively assess the associations between the TyG index and related indices (TyG-body mass index [TyG-BMI], TyG-waist circumference [TyG-WC], TyG-waist-to-height ratio [TyG-WHtR], and TyG-waist-to-hip ratio [TyG-WHR]) and the risk of incident glaucoma. We further employed Mendelian randomization (MR) analysis to assess potential causal relationships and identify mediating factors. This study rigorously evaluated the feasibility of the TyG index and related indices as biomarkers of incident glaucoma (Fig. 1).

## Methods

### Ethics statement

The UK Biobank is a prospective biomedical resource comprising approximately 500,000 participants aged 37–73 years recruited from 22 assessment centers in the United Kingdom. All study procedures followed the Declaration of Helsinki with ethical approval granted by the National Health Service Northwest Multicentre Research Ethics Committee (REC reference: 06/MRE08/65) and the National Information Governance Board for Health and Social Care [18]. Informed consent was obtained from each participant. Our study was conducted under UK Biobank application 91320, with data access compliant with the UK General Data Protection Regulation requirements. Detailed methodological protocols are publicly accessible at https://www.ukbiobank.ac.uk.

### UK Biobank study population

The prospective analysis in this study leveraged UK Biobank baseline data (2006–2010) from 503,969 UK Biobank participants. After excluding 7079 participants with glaucoma at baseline (1.4% prevalence, including 1,900 primary open-angle glaucoma [POAG], 703 primary angle-closure glaucoma [PACG], and 4,476 unspecified glaucoma), 29,841 non-European individuals, 68,220 participants with missing values of the TyG index and related indices, and 2616 participants without follow-up data or follow-up time less than 1 year, the prospective longitudinal cohort comprised 396,213 participants without glaucoma of European ancestry with complete TyG index and related index data.

### Biochemical measurements and anthropometric assessment

Blood samples were collected at baseline (2006–2010) and stored at −80 °C. Plasma levels of total triglyceride (TG, mmol/L), total cholesterol (TC, mmol/L), high-density lipoprotein (HDL, mmol/L), low-density lipoprotein (LDL, mmol/L), and glucose (Glu, mmol/L) were measured by immunoturbidimetric analysis using a Beckman Coulter AU5800 instrument. Height, weight, waist circumference, and hip circumference were measured during the initial assessment. BMI was calculated using height and weight. The TyG index and related indices were calculated as suggested before [13–15], using the following formulas:

$${\text{TyG index }} = {\text{ln[triglycerides(mg/dL) }} \times {\text{ glucose(mg/dL)/2]}},$$

$${\mathrm{TyG}} - {\text{BMI }} = {\text{TyG }} \times {\text{weight\,(kg) / [height\,(m)]}}^{{2}} ,$$

$${\mathrm{TyG}} - {\text{WC }} = {\text{TyG }} \times \,\,{\text{[waist circumference\,(cm)],}}$$

$${\mathrm{TyG}} - {\text{WHtR }} = {\mathrm{TyG}}\,{ }\, \times \,\,{\text{[waist circumference\,(cm)] / [height\,(cm)],}}$$

$${\mathrm{TyG}} - {\text{WHR }} = {\text{TyG }}\,\, \times \,\,{\text{[waist circumference\,(cm)] / [hip circumference\,(cm)]}}.$$

### Ascertainment of incident glaucoma

Participants were considered to have glaucoma if they self-reported a diagnosis of glaucoma, or a history of glaucoma surgery or laser therapy in either eye. We also included participants with the International Classification of Diseases (ICD) codes for glaucoma (ICD-9:365 [excluding 365.0]; ICD-10, H40 [excluding H40.0 and H42.0]) in the linked hospital records [19]. We excluded patients who received diagnosis before 30 years of age and control participants who reported using ocular hypertensive medication or carrying an ICD code for glaucoma suspect (ICD-9:365.0; ICD-10: H40.0). Incident glaucoma was defined as the first glaucoma diagnosis made during the follow-up. Hospital attendance data were available until October 31, 2022. Owing to variations in follow-up frequencies and medical visit intervals, the first occurrence of glaucoma during follow-up was considered the endpoint for those who developed glaucoma. The follow-up time was calculated from the baseline to the date of glaucoma diagnosis, loss to follow-up, death, or October 31, 2022, whichever occurred first [20].

### Statistical analyses

Continuous data are presented as means ± standard deviations (SDs), and categorical data as frequencies (percentages). Groups were compared using independent t-tests or chi-squared tests. The association between the TyG index (analyzed per one SD increase and in quartiles) and incident glaucoma was evaluated using multivariate Cox proportional hazards models and reported as hazard ratios (HRs) and 95% confidence intervals (CIs). Three sequential adjustment models were used: Model 1 (age and sex), Model 2 (Model 1 plus socioeconomic and lifestyle factors), and Model 3 (Model 2 plus cardiovascular and metabolic factors). Details of incident glaucoma, variables, and covariates are provided in the Supplementary Methods. Sensitivity analyses were further adjusted for or excluded participants with diabetes, hypertension, CVD, or related medications. Stratified analyses were performed according to age and sex. Non-linear relationships were examined using restricted cubic splines. Linear regression was used to assess associations of the TyG indices with corneal-compensated IOP (IOPcc) and retinal structures. Spearman’s correlation with false discovery rate (FDR) correction was used to evaluate the metabolic pathway correlations. The prediction performance was assessed using an extreme gradient boosting (XGBoost) model with hyperparameter tuning via grid search, and interpretability was explored using Shapley additive explanations (SHAP) analysis. Missing data were handled by complete-case analysis in the primary models and by k-nearest neighbor imputation in the machine-learning model. All *P* values were FDR-adjusted. All analyses were performed using R version 4.0.4 (R Foundation for Statistical Computing, Vienna, Austria).

### Mendelian randomization (MR)

MR is based on three main assumptions. First, single-nucleotide polymorphisms (SNPs) selected as instrumental variables should be robustly associated with exposure, using the TyG index. Second, genetic instruments should not be related to factors that can confound exposure-outcome associations. Third, genetic variants affect outcomes (glaucoma) only through exposure (TyG index) [21].

Genetic instruments for the TyG index were obtained from a GWAS conducted in 273,368 UK Biobank participants aged 40–69 years without diabetes or dyslipidemia [22]. Genome-wide significant SNPs associated with the TyG index were selected using a threshold of *P* < 5×10 ^−8^ and were clumped for linkage disequilibrium at R^2^ < 0.01 within a 10,000-kb window. The instrument strength was assessed using F-statistics, and only strong instruments were retained. To minimize potential horizontal pleiotropy, variants with known associations with glaucoma-related loci, diabetes, or extensive pleiotropic traits in the GWAS Catalog were excluded. Additional quality control was performed using Mendelian Randomization Pleiotropy RESidual Sum and Outlier, Steiger filtering, and leave-one-out diagnostics, to identify potential outliers or variants with evidence of reverse directionality. After these filtering steps, 55 independent SNPs were retained as instrumental variables for downstream MR analyses. Detailed instrument selection procedures, excluded variants, and SNP-level information are provided in Supplementary Methods and Supplementary Table 1.

In mediation MR, we employed two-step MR to estimate TyG-mediator-glaucoma pathways and calculated mediated proportions via coefficient product methods with delta method CIs [23]. The mediators that we evaluated included IOP [24], and retinal structural parameters [25] from the UK Biobank summary GWAS data. The analysis was performed in two steps. First, a two-sample MR was used to estimate the relationship between TyG and the potential mediator. A second, two-sample MR analysis was performed to estimate the causal effect of the mediator on glaucoma. Mediated effects were calculated using the coefficient product method with mediated proportions derived as the ratio of indirect to total effects. The CIs were computed using the delta method to establish statistical precision.

TyG-associated variants were annotated using Ensembl VEP, and corresponding genes were integrated with glaucoma GWAS and expression quantitative trait locus data using summary-data-based MR (SMR) combined with the heterogeneity in dependent instruments (HEIDI) test [26]. Genes identified by SMR and HEIDI (*P*_multi-SMR_ < 0.05 and *P*_HEIDI_ > 0.05) were further evaluated by Gene Ontology (GO) and Kyoto Encyclopedia of Genes and Genomes (KEGG) enrichment analyses to explore potential biological pathways.

## Results

### Participant characteristics

The UK Biobank analysis included 396,213 participants of European ancestry without glaucoma at the baseline. The baseline characteristics of the patients are presented in Table 1. During a mean follow-up of 8.06 ± 6.33 years, 6957 incident glaucoma cases were identified. Compared with participants who did not develop glaucoma, those who developed glaucoma were significantly older and had a markedly higher baseline IOPcc. Patients with glaucoma were more likely to be retired, less educated, more inclined to be current or previous smokers, and had a higher risk of systemic comorbidities, including hypertension, diabetes mellitus (DM), and CVD. Furthermore, the incident glaucoma group had significantly higher baseline levels of all TyG-related indices.

**Table 1:** Baseline characteristics of participants in the prospective UK Biobank cohort study

**Characteristic**	Non-incident glaucoma (n = 389,256)	Incident glaucoma (n = 6,957)	*P*
Follow-up time (years)	8.06 ± 6.33	8.16 ± 3.78	0.034
Age (years)	56.61 ± 8.03	61.59 ± 6.09	< 0.001
Sex, n (%)			0.36
Male	178,993 (46%)	3,238 (47%)	
Female	210,263 (54%)	3,719 (53%)	
Employment, n (%)			< 0.001
Employed	208,140 (53%)	2,252 (32%)	
Retired	119,885 (31%)	3,479 (50%)	
Unemployed	61,231 (16%)	1,226 (18%)	
Education, n (%)			< 0.001
Higher education	150,476 (39%)	2,350 (34%)	
Non-higher education	238,780 (61%)	4,607 (66%)	
Smoking, n (%)			< 0.001
Never	210,834 (54%)	3,548 (51%)	
Smoking	178,422 (46%)	3,409 (49%)	
Alcohol, n (%)			< 0.001
Never	25,929 (6.7%)	572 (8.2%)	
1–4 times a week	281,132 (72%)	4,794 (69%)	
> 4 times a week	81,919 (21%)	1,587 (23%)	
Drug use for lowering BP, n (%)	43,289 (11%)	1,083 (16%)	< 0.001
Drug use for lowering cholesterol, n (%)	40,357 (10%)	987 (14%)	< 0.001
Drug use for insulin, n (%)	2,283 (0.6%)	89 (1.3%)	< 0.001
CVD, n (%)	46,416 (12%)	1,459 (21%)	< 0.001
HTN, n (%)	121,764 (31%)	3,774 (54%)	< 0.001
DM, n (%)	32,423 (8.3%)	1,119 (16%)	< 0.001
Townsend deprivation index, n (%)	−1.46 ± 2.99	−1.49 ± 3.00	0.556
Index of multiple deprivation, n (%)	16.73 ± 13.68	17.12 ± 13.99	0.023
Diastolic BP (mmHg)	82.21 ± 10.66	82.18 ± 10.62	0.844
Systolic BP (mmHg)	139.81 ± 19.61	143.69 ± 19.64	< 0.001
Body mass index (kg/m^2^)	27.40 ± 4.76	27.53 ± 4.86	0.026
Fasting glucose (mmol/L)	5.11 ± 1.19	5.29 ± 1.58	< 0.001
Total cholesterol (mmol/L)	5.71 ± 1.14	5.68 ± 1.17	0.039
Triglycerides (mmol/L)	1.75 ± 1.02	1.77 ± 1.01	0.097
HDL cholesterol (mmol/L)	1.45 ± 0.38	1.47 ± 0.38	0.011
LDL cholesterol (mmol/L)	3.57 ± 0.87	3.54 ± 0.88	< 0.001
Apolipoprotein A (mmol/L)	1.54 ± 0.27	1.56 ± 0.27	< 0.001
Apolipoprotein B (mmol/L)	1.03 ± 0.24	1.03 ± 0.24	0.01
Corneal-compensated IOP (mmHg)	15.92 ± 3.64	19.94 ± 5.15	< 0.001
Height (cm)	168.73 ± 9.25	167.94 ± 9.30	< 0.001
Weight (kg)	78.24 ± 15.92	77.85 ± 15.88	0.045
Waist circumference (cm)	90.26 ± 13.47	91.49 ± 13.76	< 0.001
Hip circumference (cm)	103.40 ± 9.15	103.58 ± 9.36	0.122
Basal metabolic rate (kcal/day)	6,640.61 ± 1,371.24	6,537.85 ± 1,330.50	< 0.001
Body fat percentage (%)	31.35 ± 8.52	31.91 ± 8.53	< 0.001
Triglyceride-glucose index	8.71 ± 0.56	8.76 ± 0.57	< 0.001
TyG-body mass index	239.71 ± 49.74	242.04 ± 50.57	< 0.001
TyG-waist circumference	789.65 ± 148.03	804.15 ± 149.89	< 0.001
TyG-waist-to-height ratio	4.68 ± 0.83	4.79 ± 0.86	< 0.001
TyG-waist-to-hip ratio	7.61 ± 1.09	7.74 ± 1.09	< 0.001

*BP* = blood pressure; *CVD* = cardiovascular disease; *DM* = diabetes mellitus; *HDL* = high-density lipoprotein; *HTN* = hypertension; *LDL* = low-density lipoprotein; TyG = triglyceride-glucose

Continuous variables are expressed as means ± standard deviations, and categorical variables as numbers (percentages)

Between-group comparisons were conducted using Student’s t test or Wilcoxon rank-sum test for continuous variables, as appropriate, and the χ^2^ test for categorical variables. Incident glaucoma was defined as glaucoma newly diagnosed during follow-up among participants without glaucoma at baseline. Statistical significance was defined as a two-sided *P* < 0.05

### Associations between TyG indices and incident glaucoma

**Fig. 2:**
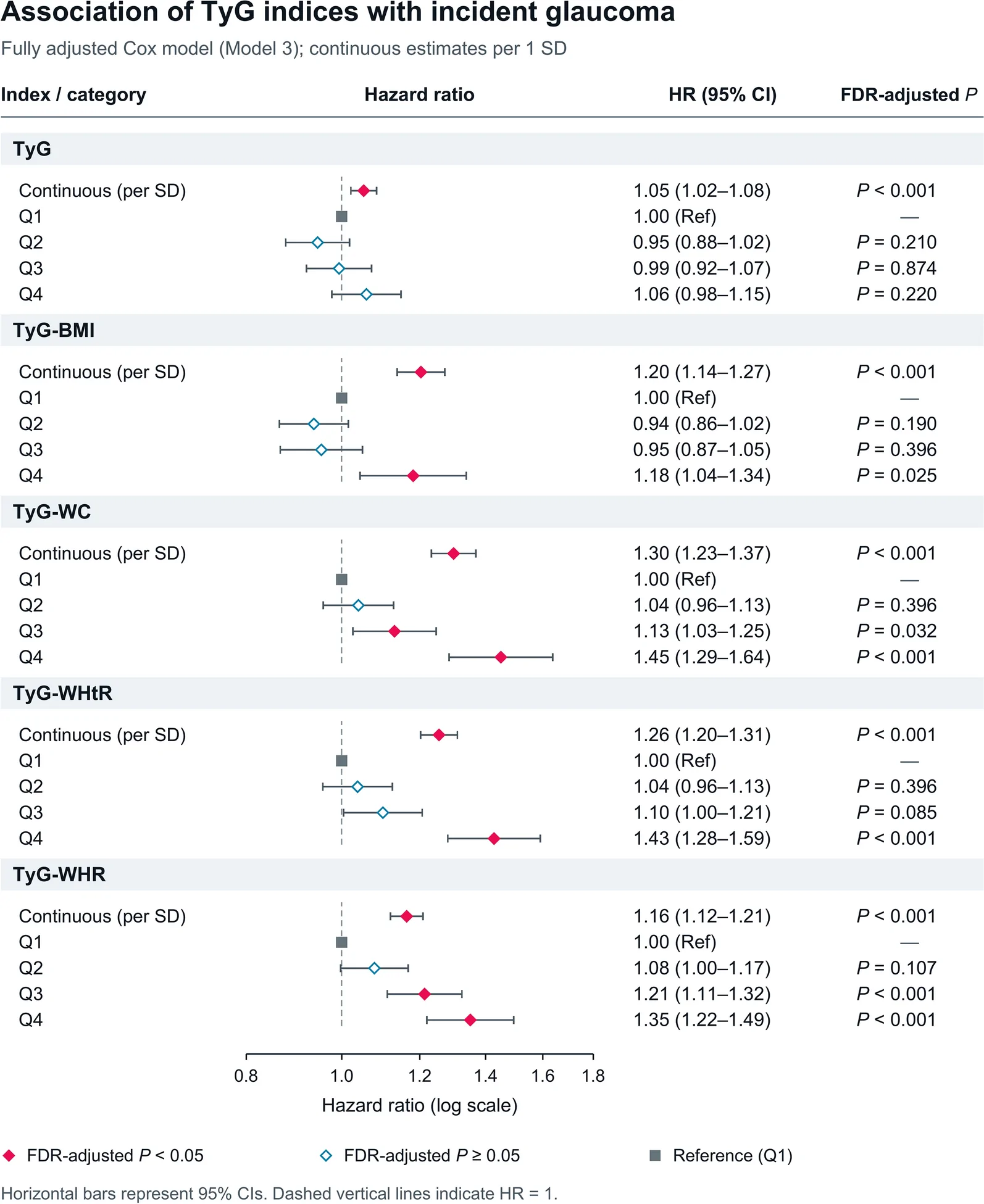
Associations of triglyceride–glucose (TyG) indices with incident glaucoma. The forest plot shows hazard ratios (HRs) and 95% confidence intervals (CIs) from the fully adjusted Cox proportional hazards model (Model 3) for the TyG index and its related indices, analyzed continuously and by quartiles. Continuous estimates are expressed per one standard deviation (SD) increase in each index. Quartiles 2–4 (Q2–Q4) are compared with the lowest quartile (Q1), which serves as the reference. Model 3 follows sequential adjustment for age, sex, socioeconomic, lifestyle, cardiovascular, and metabolic factors, as described in the Methods. Diamonds indicate HR estimates, and horizontal bars indicate 95% CIs. Filled red diamonds indicate statistically significant associations (false discovery rate [FDR]-adjusted *P* < 0.05), whereas open blue diamonds indicate associations that do not meet this threshold (FDR-adjusted *P* ≥ 0.05). Gray squares identify the reference groups (HR = 1.00); no *P* value is assigned to a reference group. The HR axis is logarithmic, and the dashed vertical lines indicate HR = 1. All displayed *P* values are FDR-adjusted. BMI, body mass index; WC, waist circumference; WHtR, waist-to-height ratio; WHR, waist-to-hip ratio. TyG-BMI, TyG-WC, TyG-WHtR, and TyG-WHR denote the TyG index multiplied by BMI, WC, WHtR, and WHR, respectively

In the primary analysis, positive associations were observed between TyG, related indices, and incident glaucoma risk. In the fully adjusted model (Model 3), each one-standard-deviation increase in the TyG index and related indices was associated with a significantly elevated hazard of glaucoma (TyG: HR = 1.05, 95% CI: 1.02–1.08, *P*_FDR_ < 0.001; TyG-BMI: HR = 1.20, 95% CI: 1.14–1.27, *P*_FDR_ < 0.001; TyG-WC: HR = 1.30, 95% CI: 1.23–1.37, *P*_FDR_ < 0.001; TyG-WHtR: HR = 1.26, 95% CI: 1.20–1.31, *P*_FDR_ < 0.001; TyG-WHR: HR = 1.16, 95% CI: 1.12–1.21, *P*_FDR_ < 0.001, Table 2 and Fig. 2). Stratified analyses by age and sex indicated significant associations between TyG indices and incident glaucoma in most subgroups, although the associations for TyG and TyG-BMI were not statistically significant among men aged ≥ 55 years (Supplementary Table 2).

**Table 2:** Association between TyG indices and incident glaucoma

TyG indices		Model 1	*P*_FDR_	Model 2	*P*_FDR_	Model 3	*P*_FDR_
Variable	Category	HR (95% CI)		HR (95% CI)		HR (95% CI)	
TyG	Continuous	1.07 (1.05–1.10)	< 0.001	1.04 (1.01–1.06)	0.005	1.05 (1.02–1.08)	< 0.001
TyG-BMI	Continuous	1.14 (1.11–1.16)	< 0.001	1.08 (1.05–1.10)	< 0.001	1.20 (1.14–1.27)	< 0.001
TyG-WC	Continuous	1.18 (1.15–1.21)	< 0.001	1.12 (1.09–1.15)	< 0.001	1.30 (1.23–1.37)	< 0.001
TyG-WHtR	Continuous	1.19 (1.17–1.22)	< 0.001	1.13 (1.10–1.16)	< 0.001	1.26 (1.20–1.31)	< 0.001
TyG-WHR	Continuous	1.19 (1.16–1.23)	< 0.001	1.13 (1.10–1.16)	< 0.001	1.16 (1.12–1.21)	< 0.001

*CI* = confidence interval; *HR* = hazard ratio; *P*_*FDR*_ = *P* value corrected for multiple comparisons using the false discovery rate method; *TyG* = triglyceride-glucose index; *TyG*-*BMI* = TyG combined with body mass index; *TyG*-*WC* = TyG combined with waist circumference; *TyG*-*WHR* = TyG combined with waist-to-hip ratio; *TyG*-*WHtR* = TyG combined with waist-to-height ratio

Sensitivity analyses demonstrated robust associations between all five TyG indices and incident glaucoma risk, after excluding participants with CVD and those using medication. However, the association for the TyG index alone was substantially attenuated and became non-significant after excluding individuals with DM (HR = 0.97, 95% CI: 0.94–1.01, *P*_FDR_ = 0.154, Supplementary Table 3) or when additionally adjusting for diabetes status in Model 3 (HR = 0.99, 95% CI: 0.96–1.02, *P*_FDR_ = 0.541, Supplementary Table 3). Furthermore, upon the exclusion of individuals with DM, consistent attenuation of the HR for incident glaucoma was observed across all TyG indices.

### Non-linear association between TyG indices and incident glaucoma

**Fig. 3:**
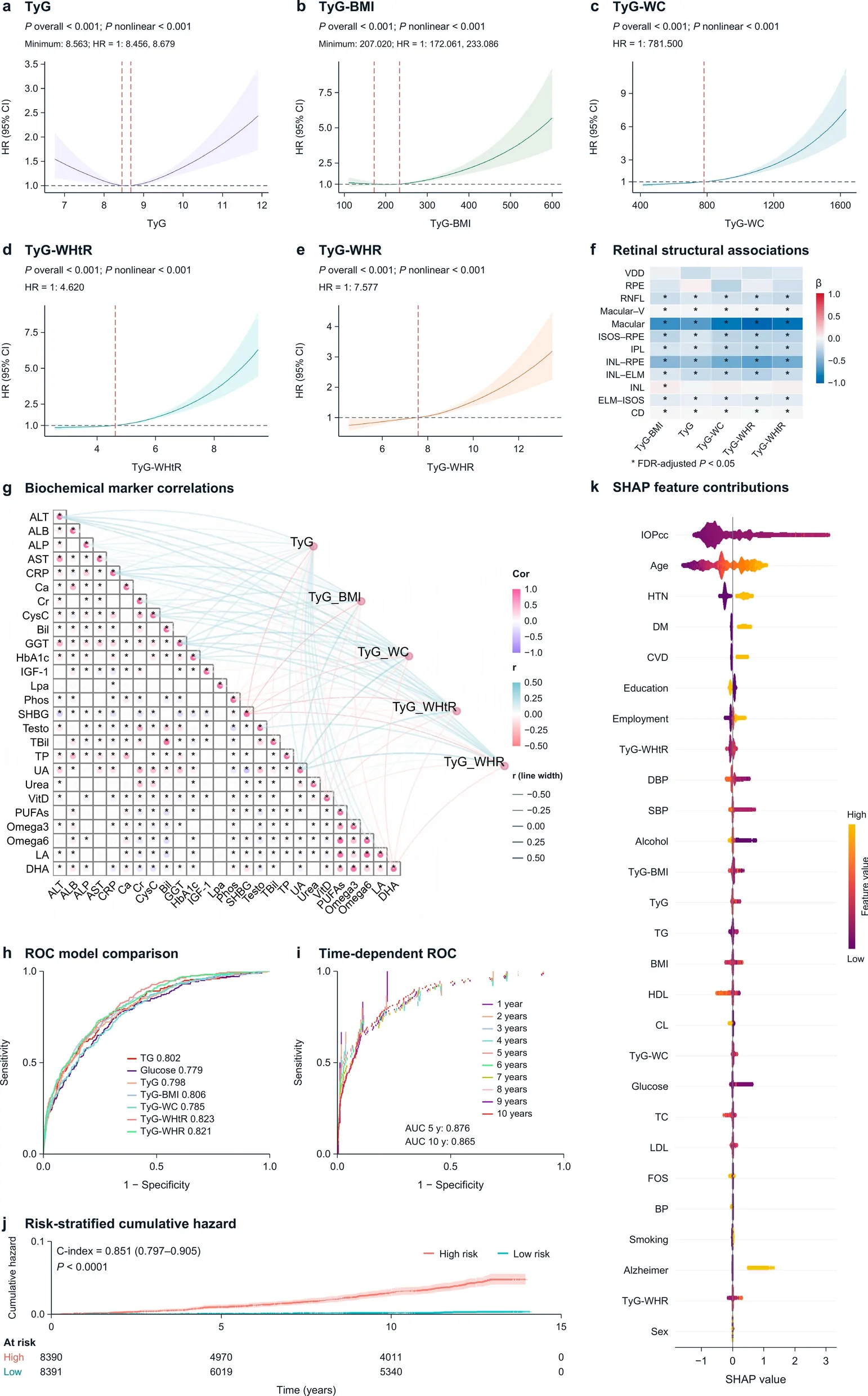
Associations of triglyceride–glucose (TyG) indices with incident glaucoma, retinal structural measurements, and biochemical markers, and performance of glaucoma prediction models. **a–e** Restricted cubic spline analyses of the TyG index (**a**), TyG-BMI (**b**), TyG-WC (**c**), TyG-WHtR (**d**), and TyG-WHR (**e**) in relation to incident glaucoma. Solid curves show estimated hazard ratios (HRs), and shaded areas show 95% confidence intervals (CIs). Horizontal dashed lines indicate HR = 1. Red vertical dashed lines mark the index values at which the fitted curve crosses HR = 1. The annotated minimum in a and b is the index value at the lowest point of the fitted curve. *P* values are shown for the overall association and nonlinearity. **f** Heatmap of regression coefficients (β) from Model 3 for associations between each TyG index and retinal measurements obtained by optical coherence tomography. Coefficients represent the change in each retinal measurement per one standard deviation increase in the corresponding TyG index. Blue indicates negative coefficients and red indicates positive coefficients; asterisks indicate false discovery rate (FDR)-adjusted *P* < 0.05. **g** Spearman correlation matrix and network for biochemical markers and TyG indices. Circle color and size indicate the direction and magnitude of pairwise correlations among biochemical markers. Network edges connect each TyG index to biochemical markers, with correlation coefficients encoded by the accompanying color and line-width keys. Asterisks mark statistically significant correlations. **h** Receiver operating characteristic (ROC) curves comparing extreme gradient boosting (XGBoost) prediction models incorporating TG, glucose, or the indicated TyG index. The numbers beside the model labels are the areas under the ROC curve (AUCs). **i–j** Time-dependent ROC curves at 1–10 years (**i**) and Kaplan–Meier curves presented on a cumulative hazard scale for high- and low-risk groups (**j**), evaluating prediction based on the TyG-WHtR model. Panel (**i**) reports the AUCs at 5 and 10 years. Panel (**j**) reports the concordance index (C-index) with its 95% CI and the *P* value for the risk-group comparison; numbers at risk are shown below the plot. **k** Shapley additive explanations (SHAP) summary plot for the XGBoost model. Each point represents a participant’s contribution for the indicated feature. The horizontal position gives the SHAP value, and color indicates the feature value from low (purple) to high (yellow). Features are ordered by their overall contribution to model predictions. ALB, albumin; ALP, alkaline phosphatase; ALT, alanine aminotransferase; AST, aspartate aminotransferase; Bil, bilirubin; BMI, body mass index; BP, blood pressure; CL, cholesterol; Cr, creatinine; CRP, C-reactive protein; CVD, cardiovascular disease; CysC, cystatin C; DBP, diastolic blood pressure; DHA, docosahexaenoic acid; DM, diabetes mellitus; ELM, external limiting membrane; FOS, fish oil supplementation; GGT, gamma-glutamyltransferase; HbA1c, glycated hemoglobin; HDL, high-density lipoprotein cholesterol; HTN, hypertension; IGF-1, insulin-like growth factor 1; INL, inner nuclear layer; IOPcc, corneal-compensated intraocular pressure; IPL, inner plexiform layer; ISOS, inner/outer photoreceptor segment junction; LA, linoleic acid; LDL, low-density lipoprotein cholesterol; Lpa, lipoprotein(a); Macular-V, macular volume; Omega3, omega-3 fatty acids; Omega6, omega-6 fatty acids; Phos, phosphate; PUFAs, polyunsaturated fatty acids; RPE, retinal pigment epithelium; RNFL, retinal nerve fiber layer; SBP, systolic blood pressure; SHBG, sex hormone-binding globulin; TBil, total bilirubin; TC, total cholesterol; Testo, testosterone; TG, triglycerides; TP, total protein; UA, urate; VDD, vertical disc diameter; VitD, vitamin D; WC, waist circumference; WHR, waist-to-hip ratio; WHtR, waist-to-height ratio; y, years

To assess the potential non-linear relationships between TyG indices and incident glaucoma, we constructed restricted cubic spline regression models based on the fully adjusted Cox Model 3. Significant non-linear associations were observed, with the TyG index exhibiting a characteristic U-shaped dose-risk relationship (Fig. 3a–e); values below 8.5 or above 8.7 were associated with increased glaucoma risk, whereas the range of 8.5–8.7 represented an interval with relatively low estimated risk (Supplementary Table 4). In subtype-specific analyses, TyG-related indices showed distinct patterns of association with POAG and PACG. For PACG, TyG, TyG-WC, and TyG-WHtR showed significant overall and significant non-linear associations. For POAG, TyG, TyG-BMI, TyG-WC, and TyG-WHtR showed significant overall associations, whereas no significant non-linear associations were detected. TyG-WHR was not significantly associated with PACG or POAG.

### Linear and correlation relationship between TyG indices and IOP, retinal structures, and biochemical markers

Linear regression analysis revealed that the continuous TyG index and its derived indices were positively associated with IOPcc. Each SD increase in these indices corresponded to an increase in IOPcc ranging from approximately 0.053 to 0.095 mmHg (Supplementary Table 5). In an additional sensitivity analysis, we adjusted for IOP in the fully adjusted model. The associations of TyG-related indices with incident glaucoma remained robust after additional IOP adjustment, including TyG-BMI, HR = 1.07, 95% CI: 1.01–1.14, *P*_FDR_ = 0.034; TyG-WC, HR = 1.09, 95% CI: 1.02–1.17, *P*_FDR_ = 0.018; and TyG-WHtR, HR = 1.11, 95% CI: 1.05–1.18, *P*_FDR_ = 0.003. Conversely, linear regression analyses indicated that higher TyG indices were inversely associated with the thickness of most retinal layers, except for the vertical disc diameter, retinal pigment epithelium, and inner nuclear layer. The strongest negative association was observed for macular thickness (Supplementary Table 6 and Fig. 3f). However, the proportion of the variance explained (R^2^) for these associations was relatively low, necessitating cautious interpretation. Furthermore, TyG indices showed robust positive correlations with biomarkers of liver function and inflammation, including alanine aminotransferase, C-reactive protein, gamma-glutamyl transferase, and urate, and significant negative correlations with sex hormone-binding globulin, vitamin D, and docosahexaenoic acid levels (Supplementary Table 7 and Fig. 3g).

The XGBoost machine learning model of the TyG index and related indices demonstrated a robust predictive performance for incident glaucoma (Fig. 3h–k). The TyG-WHtR achieved the highest predictive ability, with a C-index of 0.851 (95% CI: 0.797–0.905, Supplementary Table 8 and Fig. 3j) and an area under the curve (AUC) of 0.823. SHAP analysis (Fig. 3k) also showed that the TyG-WHtR was the most important factor among the TyG indices. Predictive performance varied across the composite indices; TyG-WHtR had the highest AUC (0.823), followed by TyG-WHR (0.821), in Supplementary Table 8.

### MR analysis of TyG index and glaucoma

We retained 55 independent TyG-associated SNPs as instrumental variables, all with F-statistics >10 (range, 29.8–912.0). In the primary inverse-variance weighted (IVW) analysis, genetically predicted higher TyG index showed a nominal association with increased glaucoma risk (odds ratio [OR] = 1.27, 95% CI: 1.00–1.62, *P* = 0.046; Supplementary Table 9 and Supplementary Figs. 1 and 2). The estimates from alternative MR methods were not statistically significant. No evidence of directional horizontal pleiotropy based on the MR-Egger intercept was identified (intercept = 0.0052, *P* = 0.178), although heterogeneity was observed across instruments (Cochran’s Q = 74.41, *P* = 0.034). Leave-one-out analysis did not identify any single influential SNP. Therefore, these findings were interpreted as suggestive genetic evidence rather than conclusive evidence of causality.

The diabetes-related MR and mediation analyses are presented in Supplementary Tables 10 and 11. In exploratory mediation MR analyses, genetically predicted TyG was associated with vertical cup-to-disc ratio (VCDR) (β = 0.16, *P* = 0.032), and genetically predicted VCDR was associated with glaucoma risk (β = 0.42, *P* = 5.96 × 10⁻⁷), corresponding to an estimated mediated proportion of 28.0% (Supplementary Tables 12 and 13). By contrast, genetically predicted TyG was not associated with IOP (OR = 1.05, 95% CI: 0.94–1.18, *P* = 0.376; Supplementary Table 14).

Functional annotation and SMR analyses were performed to determine the biological context of TyG-related genetic signals. TyG-associated SNPs were annotated to 136 genes using Ensembl, of which 27 showed evidence of association with glaucoma in SMR and HEIDI analyses. GO and KEGG enrichment analyses highlighted pathways related to carbohydrate, hexose, and glucose metabolism; oxidoreductase activity; and cholesterol metabolism.

## Discussion

In this large prospective analysis of UK Biobank participants, higher TyG-related indices were associated with an increased risk of glaucoma. Among these indices, adiposity-integrated TyG measures, particularly TyG-WC and TyG-WHtR, showed stronger associations than TyG index alone. The TyG index also demonstrated a non-linear association with glaucoma risk, with a higher estimated risk at both lower and higher extremes. These observational findings were complemented by MR analyses, which provided suggestive genetic evidence consistent with a potential causal relationship between higher TyG levels and glaucoma risk. However, the MR results should be interpreted cautiously, given the borderline IVW estimate, non-significant sensitivity estimates, and evidence of instrument heterogeneity. Overall, systemic glucose-lipid metabolic dysregulation may contribute to glaucoma susceptibility and that TyG-related indices may serve as accessible metabolic indicators for glaucoma risk stratification.

The TyG index is a low-cost surrogate marker of insulin resistance that integrates circulating triglyceride and glucose levels [22]. It has been widely investigated in cardiometabolic disorders, including type 2 diabetes, CVD metabolic dysfunction-associated steatotic liver disease, and diabetic retinopathy [13–15, 23, 27]. Our study extends this evidence to glaucoma by evaluating TyG-related indices in a prospective setting and by incorporating genetic analyses. Importantly, the association between TyG index alone and incident glaucoma was modest, whereas indices combining TyG with anthropometric measures showed stronger associations. This pattern suggests that the glaucoma-related metabolic burden may not be fully captured by glucose lipid status alone. Instead, central adiposity and body fat distribution may provide additional information regarding the systemic metabolic stress, inflammation, vascular dysfunction, and obesity-related biological processes associated with glaucomatous optic neuropathy [28].

From a clinical perspective, these findings should be interpreted as evidence of risk stratification rather than a diagnosis. The observed effect size of the TyG index alone was small, and TyG-related indices should not be considered standalone screening or diagnostic tools for glaucoma. However, their accessibility and low cost may make them useful as adjunctive metabolic indicators, particularly among individuals with adverse cardiometabolic profiles or other established glaucoma risk factors [22]. The stronger performance of TyG-WC and TyG-WHtR also suggests that central adiposity may be more informative than general adiposity for identifying individuals with higher metabolic vulnerability [29]. Nevertheless, before clinical implementation, these indices require validation in independent cohorts, assessment of calibration and incremental predictive value, and decision curve analyses to determine whether they improve clinical risk prediction beyond established factors such as age, IOP, family history, myopia, and optic nerve parameters [30].

Several biological pathways may explain the association between the TyG-related indices and glaucoma risk. Insulin resistance and glucose-lipid metabolic dysfunction can promote endothelial dysfunction, oxidative stress, chronic low-grade inflammation, and mitochondrial impairment [31, 32]. These processes are highly relevant to glaucoma because the optic nerve head and RGCs are metabolically active tissues that are vulnerable to impaired perfusion, ischemic stress, and oxidative injury [33, 34]. Hyperglycemia may increase mitochondrial reactive oxygen species production, advanced glycation end-product accumulation, and protein kinase C activation, while dyslipidemia may promote lipid oxidation and inflammatory signaling [35]. Together, these pathways can impair nitric oxide bioavailability, disrupt vascular autoregulation, and compromise ocular microcirculation [36, 37]. Such vascular and metabolic stress may reduce the resilience of RGCs to intraocular pressure-related injury and contribute to glaucomatous neurodegeneration [2, 38].

The associations between incident glaucoma and TyG-BMI, TyG-WC, and TyG-WHtR persisted after additional adjustments for IOP, suggesting that IOP is unlikely to fully explain these associations. Although TyG-related indices were statistically associated with higher IOPcc in observational analyses, the effect sizes were very small, with each SD increase corresponding to only a 0.053–0.095 mmHg difference in IOPcc. Moreover, MR mediation did not support IOP as a major mediator of the TyG–glaucoma association. These findings suggest that TyG-related metabolic dysfunction may be linked to glaucoma partly through IOP-independent mechanisms, such as vascular dysregulation, impaired optic nerve head perfusion, neuroinflammation, mitochondrial dysfunction, and RGC vulnerability [34, 39].

The non-linear association observed for the TyG index further complicates the interpretation of metabolic risk. Higher TyG values are biologically consistent with insulin resistance, hypertriglyceridemia, and impaired glucose metabolism [22, 32]. By contrast, the increased risk observed with lower TyG values may reflect heterogeneous conditions rather than the direct harmful effect of low TyG levels. Low TyG may be associated with frailty, undernutrition, chronic illness, medication use, or other systemic factors that influence glaucoma risk or healthcare contact [40]. Therefore, the U-shaped pattern should be interpreted cautiously and requires replication. Rather than defining a clinically “unsafe” TyG range, these findings suggest that both extremes of metabolic status may identify individuals with distinct systemic risk profiles relevant to ocular health [41].

Subtype-specific analyses suggest that the association between TyG-related indices and glaucoma may vary between POAG and PACG. For PACG, several TyG-related indices showed significant overall and non-linear associations, whereas for POAG, the associations were mainly overall, without significant non-linear components. These findings should be considered exploratory because the glaucoma subtype classification in the UK Biobank is based on available diagnostic records rather than standardized gonioscopy or uniform ophthalmic examination of all participants [20]. Nonetheless, subtype heterogeneity is biologically plausible. PACG is strongly influenced by anterior segment anatomy, aqueous humor dynamics, and pressure-related mechanisms, whereas POAG involves a broader combination of genetic susceptibility, vascular dysregulation, neurodegeneration, and IOP-related injury [1, 42]. Metabolic dysfunction and central adiposity may therefore influence different glaucoma subtypes through partially distinct pathways [10]. Larger studies with standardized ophthalmic phenotyping are needed to confirm these subtype-specific patterns.

MR analyses provided additional, but not definitive, evidence to support a potential link between TyG and glaucoma. The primary IVW estimate suggested that genetically predicted higher TyG levels were associated with an increased glaucoma risk. However, the association was borderline, alternative MR methods were not statistically significant, and heterogeneity was detected across the instruments. These features preclude strong causal inference [43]. The MR results should therefore be considered suggestive genetic evidence that complements observational findings rather than as proof of causality. Exploratory mediation MR analyses further suggested that DM and VCDR may partly contribute to the TyG-glaucoma relationship. This is consistent with prior evidence linking diabetes and metabolic risk factors with glaucoma and optic disc cupping [44]. However, mediation estimates from summary-level MR require cautious interpretation, particularly when mediators are biologically correlated and when outcome phenotyping is heterogeneous.

Exploratory functional annotation and pathway analyses provided biological context for the observed associations. TyG-related variants mapped to genes implicated in energy metabolism, lipid-related metabolic processes, retinol metabolism, glycerophospholipid metabolism, and transcriptional or epigenetic regulation [45]. These pathways are relevant to retinal homeostasis, mitochondrial function, oxidative stress responses, and neuronal membrane integrity [33, 34, 46]. However, given the limited number of SMR-significant genes and the exploratory nature of the enrichment analyses, these results should not be interpreted as mechanistic evidence. Instead, they hypothesized that metabolic and transcriptional regulatory pathways connect systemic insulin resistance to glaucomatous neurodegeneration.

This study has several strengths, including its large prospective design, long-term follow-up, assessment of multiple TyG-related indices, integration of ocular phenotypes, subtype analyses, and a complementary MR framework. However, this study has some limitations. First, the participants were predominantly middle-aged and older individuals of European ancestry from the UK Biobank, which may limit generalizability to younger populations and other ethnic groups. Second, glaucoma ascertainment was based on self-reported diagnoses, glaucoma-related procedures, and linked hospital diagnostic codes, rather than standardized ophthalmic examinations for all participants. Although prevalent and suspected glaucoma were excluded wherever possible, outcome misclassification was possible. The subtype classification for POAG and PACG may also be imperfect because gonioscopy and uniform anterior segment assessment were not available for all participants. Third, residual confounding factors cannot be fully excluded. Important factors, such as systemic or ocular steroid use, refractive status, myopia, family history of glaucoma, ocular anatomy, and healthcare utilization, were not fully available. Fourth, detection bias may have influenced the observational findings, as individuals with metabolic disorders may have had more frequent healthcare contact and a higher likelihood of receiving an ophthalmic diagnosis. Complementary MR analysis is less susceptible to conventional confounding but does not directly eliminate detection bias in the observational UK Biobank analysis. Fifth, TyG-related indices were calculated from baseline measurements only. Longitudinal changes in glucose and triglyceride levels, anthropometric measures, medication use, and metabolic health were not captured. Sixth, although the MR instruments showed adequate strength, the borderline IVW estimate, non-significant sensitivity methods, and instrument heterogeneity warrant cautious interpretation. Finally, although machine learning analyses showed good discrimination within the UK Biobank, external validation, calibration assessment, and decision curve analysis are required before the clinical utility of TyG-related indices can be established.

## Conclusions

TyG-related indices, particularly adiposity-integrated measures, were associated with an increased risk of glaucoma. These findings supported the potential role of systemic glucose-lipid metabolic dysfunction and central adiposity in glaucoma susceptibility, possibly through vascular, inflammatory, oxidative, and neurodegenerative pathways. TyG-related indices might serve as accessible metabolic indicators for glaucoma risk stratification rather than standalone diagnostic tools. Further studies with repeated metabolic measurements, standardized ophthalmic phenotyping, diverse populations, and interventional designs are needed to determine whether modifying the TyG-related metabolic status can reduce glaucoma risk.

## Supplementary Information

Supplementary Material 1

## Data Availability

The UK Biobank data used in this study were accessed under Application No. 91320 and are available to approved researchers after successful application through the UK Biobank access process (https://www.ukbiobank.ac.uk/enable-your-research/apply-for-access). The GWAS summary statistics for retinal structures are available in Zenodo under accession code 11217687 (https://zenodo.org/records/11217687). GWAS summary statistics for diabetes and intraocular pressure are available from the GWAS Catalog under accession codes GCST90468124 and GCST90309360, respectively. All other data supporting the findings are included in this article and its supplementary materials.

## Abbreviations

AUC: Area under the curve
BMI: Body mass index
CI: Confidence interval
CVD: Cardiovascular disease
DM: Diabetes mellitus
FDR: False discovery rate
GO: Gene Ontology
GWAS: Genome-wide association study
HDL: High-density lipoprotein
HEIDI: Heterogeneity in dependent instruments
HR: Hazard ratio
ICD: International classification of diseases
IOP: Intraocular pressure
IOPcc: Corneal-compensated intraocular pressure
IVW: Inverse-variance weighted
KEGG: Kyoto Encyclopedia of Genes and Genomes
LDL: Low-density lipoprotein
MR: Mendelian randomization
OR: Odds ratio
PACG: Primary angle-closure glaucoma
POAG: Primary open-angle glaucoma
RGC: Retinal ganglion cell
ROC: Receiver operating characteristic
SD: Standard deviation
SHAP: Shapley additive explanations
SMR: Summary-data-based Mendelian randomization
SNP: Single-nucleotide polymorphism
TG: Triglyceride
TyG: Triglyceride-glucose index
TyG-BMI: TyG-body mass index
TyG-WC: TyG-waist circumference
TyG-WHR: TyG-waist-to-hip ratio
TyG-WHtR: TyG-waist-to-height ratio
VCDR: Vertical cup-to-disc ratio
VEP: Variant Effect Predictor
XGBoost: Extreme gradient boosting
