# Supplementary material for: Association between triglyceride-glucose indices and incident glaucoma: integrated UK Biobank and Mendelian randomization study
Source: Eye Vis (Lond). 2026 Oct 10;13:43. doi: 10.1186/s40662-026-00516-x (PMC13653385; doi:10.1186/s40662-026-00516-x)
Supplement: Supplementary file 1 [file 40662_2026_516_MOESM1_ESM.docx]

**Supplementary Materials**

**Contents**

**Supplementary Methods**

Ascertainment of Incident Glaucoma, Variables and Covariates	2

XGBoost Machine Learning Prediction Model	4

Mendelian Randomization	5

**Supplementary Tables**

Supplementary Table 1. Instrumental variables for the Mendelian randomization analysis between TyG index and glaucoma	7

Supplementary Table 2. Age and sex stratified analysis of association between TyG index and related indices and incident glaucoma	10

Supplementary Table 3. Sensitivity analysis of association between TyG index and related indices and incident glaucoma	11

Supplementary Table 4. Association analysis of high/low levels of TyG index and related indices and incident glaucoma	13

Supplementary Table 5. Linear association analysis of IOPcc and TyG	14

Supplementary Table 6. Linear association analysis of retinal structures and TyG indices	15

Supplementary Table 7. Correlation analysis of biochemical markers and TyG in glaucoma	18

Supplementary Table 8. XGBoost prediction model for incident glaucoma	21

Supplementary Table 9. Mendelian randomization analysis of TyG and glaucoma	22

Supplementary Table 10. Mendelian randomization analysis of TyG and DM	23

Supplementary Table 11. Mediation mendelian randomization of TyG, DM and glaucoma	24

Supplementary Table 12. Mendelian randomization of retinal structures and glaucoma	25

Supplementary Table 13. Mediation mendelian randomization of TyG, VCDR and glaucoma	27

Supplementary Table 14. Mendelian randomization analysis of TyG and IOP	28

**Supplementary Figures**

Supplementary Figure 1. Mendelian randomization of forest plot with TyG and glaucoma	29

Supplementary Figure 2. Mendelian randomization of scatter plot with TyG and glaucoma	30

**Supplementary Methods**

**Ascertainment of Incident Glaucoma**

Participants were considered to have glaucoma if they self-reported a diagnosis of glaucoma or a history of glaucoma surgery or laser therapy in either eye. We also included participants carrying an International Classification of Diseases (ICD) code for glaucoma (ICD-9: 365.* [excluding 365.0]; ICD-10: H40.* [excluding H40.0 and H42.*]) in the linked hospital records. We excluded patients who received a diagnosis before 30 years of age and control participants who reported using ocular hypertensive medication or carrying an ICD code for glaucoma suspect (ICD-9: 365.0; ICD-10: H40.0). Incident glaucoma was defined as the first diagnosis of glaucoma during follow-up. Hospital attendance data were available up to October 31, 2022. Due to the variations in follow-up frequencies and medical visit intervals, the first occurrence of glaucoma during follow-ups was considered the endpoint for those who developed glaucoma. Follow-up time was calculated from baseline to the date of glaucoma diagnosis, loss to follow-up, death, or October 31, 2022, whichever occurred first.

**Ascertainment of Variables and Covariates**

The intraocular pressure (IOP) was measured once for each eye using the ocular response analyzer (ORA) non-contact tonometer (Reichert Corp). The ORA flattens the cornea with a jet of air, causing an initial inward applanation, followed by an outward applanation event as the cornea returns to its original shape. An electro-optical system measures the air pressures at the initial inward applanation and the outward applanation event and combines them linearly to derive an IOP that accounts for corneal biomechanical properties (IOPcc). Participant-level IOP was calculated as the average of both eyes or as either right or left eye value if data were available for only one eye.

Retinal optical coherence tomography (OCT) data (Data-Field 28520) were acquired in 2012 using the TOPCON 3D OCT 1000 Mk2 with Topcon Advanced Boundary Segmentation (TABS) software. The OCT system performed 3D retinal scans and fundus photography. We included eight parameters that are clinically relevant to glaucoma and demonstrated minimal missing values: vertical cup-to-disc ratio (C/D), average retinal nerve fiber layer (RNFL) thickness, overall macular thickness (macular), overall average retinal pigment epithelium thickness (RPE), average ganglion cell-inner plexiform layer (GCIPL) thickness, average thickness measured between the external limiting membrane (ELM) to the inner and outer photoreceptor segments (ISOS), average thickness measured between the inner nuclear layer (INL) to the RPE，average thickness measured between the ISOS to the RPE.

A series of demographic, socioeconomic, and behavioral factors were included as covariates in the analysis: age, sex, employment, education, smoking, alcohol consumption, Townsend deprivation index (TDI), body mass index (BMI), systolic and diastolic blood pressure (SBP, DBP), total cholesterol (TC), triglycerides (TG), high-density lipoprotein (HDL), low-density lipoprotein (LDL), fasting blood glucose (FBG), diabetes mellitus (DM), hypertension, and cardiovascular disease (CVD). Participants answered detailed touch-screen questionnaires that cover a wide range of demographic, health, and lifestyle information. The choices for ethnicity included White (English/Irish or other White background), Asian or British Asian (Indian/Pakistani/Bangladeshi or other Asian background), Black or Black British (Caribbean, African, or other Black background), Chinese, mixed (White and Black Caribbean or African, White and Asian, or other mixed background), or another ethnic group (not defined). We subsequently combined ethnicity groups into European and non-European ancestry. Smoking status was determined by self-report and was subsequently combined into ever smoker or never smoker. Alcohol status was determined by self-report and was combined into never, 1 to 4 times a week, or more than 4 times a week. Employment was categorized as “Employed” (in paid employment or self-employed), “Retired”, and “Unemployed”. Education was categorized as “Higher Education” (College or University degree, or other professional qualification), “Non-higher Education”. Townsend deprivation index calculated immediately prior to participant joining UK Biobank. Based on the preceding national census output areas. Each participant is assigned a score corresponding to the output area in which their postcode is located. Blood pressure was measured twice using the HEM-70151T digital blood pressure monitor (Omron), and the mean was used in the analysis. Weight was measured with the BV-418 MA body composition analyzer (Tanita). Height was measured using a Seca 202 height measure (Seca). BMI was calculated as weight (kg)/height (m²). Blood samples were collected at baseline (2006–2010) and stored at −80°C. Plasma levels of total triglycerides (TG, mmol/L), total cholesterol (TC, mmol/L), high-density lipoprotein (HDL, mmol/L), low-density lipoprotein (LDL, mmol/L), and glucose (Glu, mmol/L) were measured by immunoturbidimetric analysis on a Beckman Coulter AU5800. Height, weight, and waist were measured during the initial Assessment Centre visit. BMI value is constructed from height and weight. Diabetes status was defined by self-report of DM and hospital records (ICD-9: 250, and ICD-10: E10 – E14) or use of insulin. Hypertension status was defined by self-report of hypertension and hospital records (ICD-9: 401 – 405, ICD-10: I10 – I13, I15, and O10) or use of anti - hypertensive medication. Cardiovascular diseases (CVD) status was defined by self-report and hospital records (ICD-9: 4140, 4148, 4149, 410–412, and ICD-10: I20–25).

**XGBoost Machine Learning Prediction Model**

An extreme gradient boosting (XGBoost) machine learning framework evaluated TyG indices predictive capacity for incident glaucoma risk, addressing missing value imputation through k-nearest neighbors (KNN) algorithms based on Euclidean distance matrices. Feature selection from 26 demographic, lifestyle, biomarker, and PRS variables employed. Following 8:2 training-test partitioning, hyperparameter optimization used grid search with model interpretability enhanced through Shapley Additive Explanations (SHAP). Performance assessment incorporated survival analytics, Kaplan–Meier (KM) risk stratification, and time-dependent area under receiver operating characteristic curve (time-ROC) metrics.

**Mendelian Randomization (MR)**

In a genome-wide association studies (GWAS) of the TyG index conducted within the UK Biobank cohort comprising 273,368 participants aged 40–69 years without diabetes or dyslipidemia, 192 potential instrumental variables for TyG were initially identified based on thresholds of *P* < 5 × 10⁻⁸ and R² < 0.01. To ensure the reliability of MR estimates, these instruments underwent rigorous filtering. First, single nucleotide polymorphisms (SNPs) were retained based on an F-statistic > 30 and linkage disequilibrium pruning (R² < 0.01 within a 10,000 kb window), yielding 108 SNPs. Second, one SNP located in the glaucoma-associated gene ABCA1 (rs2244278) was excluded, along with 23 SNPs (rs10811661, rs2925979, rs58542926, rs116843064, rs878521, rs6066138, rs38205, rs6073958, rs6547692, rs72836561, rs10260148, rs13108218, rs139974673, rs1760801, rs1801689, rs185139895, rs2035816, rs2081687, rs2277844, rs6710938, rs67981690, rs7140110, rs852424, rs72801474) that showed significant associations with both TyG and diabetes, and five SNPs (rs1532085, rs4722551, rs483082, rs1471251, rs99780) reported in the GWAS catalog to be associated with more than 100 traits at *P* < 5 × 10⁻⁸, indicating substantial horizontal pleiotropy. This left 60 SNPs for further analysis. Subsequently, MR-PRESSO, Steiger filtering, and leave-one-out sensitivity analyses were performed, leading to the removal of five outlier SNPs (rs13074711, rs4921914, rs10889332, rs188247550, rs17321515) due to directional pleiotropy, heterogeneity, or outlier characteristics. The final set of 55 rigorously selected, independent, and exclusively TyG-associated instrumental variables was used in downstream MR analyses. Notably, an initial MR analysis using all 192 SNPs suggested a significant association between TyG and glaucoma risk; however, considerable heterogeneity and horizontal pleiotropy undermined the reliability of these results. This underscored the necessity of stringent instrument selection, particularly as subsequent analyses identified diabetes as a key mediating factor in the pathway from TyG to glaucoma.

The GWAS for retinal structures were derived from the UK Biobank cohort (n=60,748 individuals of white British ancestry). The SNP-based heritability (h²) for the 46 OCT measures ranged from 9.28% to 68.28%, all of which passed a 5% false discovery rate threshold. The GWAS summary statistics are available in the Zenodo database under accession code 11217687. The 46 OCT-derived traits included bilateral measurements of the RNFL, GCIPL, and other retinal layers, along with sectoral measurements and six optic disc metrics (for the left eye only, including vertical cup-to-disc ratio). Additionally, the summary statistics for the diabetes and IOP GWAS were obtained from the GWAS Catalog (Diabetes: GCST90468124, IOP: GCST90309360), which were based on 394,626 individuals and 77,267of European ancestry from the UK Biobank.

**Supplementary Tables**

Supplementary Table 1. Instrumental variables for the Mendelian randomization analysis between TyG and glaucoma

|  |  |  |  |  |  | Exposure |  |  | Outcome |  |  |  |  |
| --- | --- | --- | --- | --- | --- | --- | --- | --- | --- | --- | --- | --- | --- |
| No. | Exposure | Outcome | SNP | EA | OA | β | SE | *P* | β | SE | *P* | F | Steiger |
| 1 | TyG | Glaucoma | rs10783828 | A | G | 9.04E−03 | 1.47E−03 | 9.04E−10 | 1.12E−02 | 9.29E−03 | 2.29E−01 | 3.75E+01 | 2.54E−05 |
| 2 | TyG | Glaucoma | rs10786069 | C | T | 1.31E−02 | 1.38E−03 | 2.06E−21 | −6.78E−03 | 9.22E−03 | 4.62E−01 | 9.03E+01 | 5.84E−13 |
| 3 | TyG | Glaucoma | rs10832027 | G | A | −1.23E−02 | 1.48E−03 | 1.37E−16 | 3.73E−03 | 9.22E−03 | 6.86E−01 | 6.84E+01 | 1.48E−10 |
| 4 | TyG | Glaucoma | rs10861679 | C | T | 9.35E−03 | 1.51E−03 | 5.35E−10 | −1.70E−02 | 1.09E−02 | 1.18E−01 | 3.85E+01 | 4.81E−05 |
| 5 | TyG | Glaucoma | rs11075253 | A | C | −1.41E−02 | 1.50E−03 | 5.65E−21 | −1.65E−02 | 1.02E−02 | 1.06E−01 | 8.83E+01 | 4.22E−11 |
| 6 | TyG | Glaucoma | rs11118610 | C | A | −9.03E−03 | 1.39E−03 | 8.09E−11 | −8.79E−03 | 9.20E−03 | 3.39E−01 | 4.22E+01 | 3.19E−06 |
| 7 | TyG | Glaucoma | rs11134475 | A | G | −1.69E−02 | 1.42E−03 | 1.15E−32 | −2.21E−03 | 9.76E−03 | 8.21E−01 | 1.42E+02 | 3.82E−21 |
| 8 | TyG | Glaucoma | rs11216236 | T | C | 2.40E−02 | 3.42E−03 | 2.21E−12 | −1.68E−02 | 2.99E−02 | 5.74E−01 | 4.93E+01 | 1.09E−07 |
| 9 | TyG | Glaucoma | rs113344423 | A | G | 2.13E−02 | 3.02E−03 | 1.70E−12 | 2.18E−02 | 1.62E−02 | 1.79E−01 | 4.98E+01 | 1.08E−06 |
| 10 | TyG | Glaucoma | rs11651957 | A | G | 1.86E−02 | 2.94E−03 | 2.14E−10 | −1.86E−02 | 1.73E−02 | 2.83E−01 | 4.03E+01 | 7.88E−06 |
| 11 | TyG | Glaucoma | rs11657238 | A | G | −7.85E−03 | 1.38E−03 | 1.41E−08 | −5.52E−03 | 9.32E−03 | 5.54E−01 | 3.22E+01 | 2.56E−05 |
| 12 | TyG | Glaucoma | rs12173130 | C | T | 9.71E−03 | 1.77E−03 | 4.07E−08 | 4.22E−03 | 1.12E−02 | 7.06E−01 | 3.01E+01 | 2.80E−05 |
| 13 | TyG | Glaucoma | rs12446515 | T | C | −1.88E−02 | 1.47E−03 | 4.76E−37 | −4.85E−03 | 1.03E−02 | 6.36E−01 | 1.62E+02 | 2.62E−23 |
| 14 | TyG | Glaucoma | rs12610709 | A | G | 1.40E−02 | 1.83E−03 | 2.24E−14 | 6.29E−03 | 1.29E−02 | 6.25E−01 | 5.83E+01 | 4.93E−09 |
| 15 | TyG | Glaucoma | rs12885801 | A | C | 9.09E−03 | 1.62E−03 | 2.11E−08 | −6.48E−03 | 1.07E−02 | 5.45E−01 | 3.14E+01 | 3.39E−05 |
| 16 | TyG | Glaucoma | rs12934528 | C | T | 1.35E−02 | 1.96E−03 | 5.15E−12 | −2.42E−03 | 1.52E−02 | 8.74E−01 | 4.76E+01 | 4.89E−08 |
| 17 | TyG | Glaucoma | rs13289566 | T | C | −1.18E−02 | 1.67E−03 | 1.37E−12 | 2.67E−03 | 1.12E−02 | 8.13E−01 | 5.02E+01 | 2.72E−08 |
| 18 | TyG | Glaucoma | rs1585705 | C | A | 8.77E−03 | 1.50E−03 | 4.64E−09 | 2.41E−02 | 9.66E−03 | 1.27E−02 | 3.43E+01 | 1.24E−03 |
| 19 | TyG | Glaucoma | rs17145750 | T | C | −5.61E−02 | 1.86E−03 | 5.28E−200 | −1.09E−02 | 1.29E−02 | 3.98E−01 | 9.12E+02 | 6.95E−125 |
| 20 | TyG | Glaucoma | rs1716407 | G | A | −1.51E−02 | 1.40E−03 | 4.90E−27 | −1.50E−02 | 9.31E−03 | 1.07E−01 | 1.16E+02 | 1.35E−14 |
| 21 | TyG | Glaucoma | rs17656269 | T | C | 8.75E−03 | 1.47E−03 | 2.65E−09 | −3.92E−04 | 9.49E−03 | 9.67E−01 | 3.54E+01 | 1.92E−06 |
| 22 | TyG | Glaucoma | rs17694506 | C | T | 9.00E−03 | 1.42E−03 | 2.19E−10 | 1.41E−02 | 9.60E−03 | 1.42E−01 | 4.03E+01 | 2.33E−05 |
| 23 | TyG | Glaucoma | rs186696265 | T | C | −4.73E−02 | 5.93E−03 | 1.51E−15 | −3.98E−02 | 3.66E−02 | 2.78E−01 | 6.36E+01 | 7.96E−09 |
| 24 | TyG | Glaucoma | rs2302883 | C | T | 8.86E−03 | 1.62E−03 | 4.71E−08 | 4.71E−03 | 1.31E−02 | 7.18E−01 | 2.98E+01 | 2.94E−05 |
| 25 | TyG | Glaucoma | rs2745400 | A | G | 8.26E−03 | 1.37E−03 | 1.76E−09 | 8.85E−03 | 9.31E−03 | 3.42E−01 | 3.62E+01 | 1.91E−05 |
| 26 | TyG | Glaucoma | rs2943645 | C | T | −2.09E−02 | 1.43E−03 | 2.40E−48 | −1.07E−02 | 9.55E−03 | 2.64E−01 | 2.14E+02 | 1.46E−28 |
| 27 | TyG | Glaucoma | rs3025053 | A | G | −1.35E−02 | 2.12E−03 | 2.40E−10 | 8.90E−03 | 1.41E−02 | 5.27E−01 | 4.01E+01 | 2.40E−06 |
| 28 | TyG | Glaucoma | rs3120619 | A | G | 1.19E−02 | 1.80E−03 | 4.45E−11 | −6.66E−03 | 1.26E−02 | 5.96E−01 | 4.34E+01 | 6.27E−07 |
| 29 | TyG | Glaucoma | rs34820917 | A | G | −1.58E−02 | 2.85E−03 | 2.97E−08 | 6.71E−03 | 1.70E−02 | 6.93E−01 | 3.07E+01 | 2.41E−05 |
| 30 | TyG | Glaucoma | rs35750610 | C | T | 1.85E−02 | 2.35E−03 | 3.09E−15 | −2.03E−02 | 1.70E−02 | 2.33E−01 | 6.22E+01 | 1.76E−08 |
| 31 | TyG | Glaucoma | rs3750571 | A | C | −1.24E−02 | 1.90E−03 | 6.27E−11 | −9.33E−03 | 1.32E−02 | 4.79E−01 | 4.27E+01 | 1.32E−06 |
| 32 | TyG | Glaucoma | rs3808976 | G | A | 9.82E−03 | 1.70E−03 | 7.84E−09 | −9.65E−03 | 1.03E−02 | 3.47E−01 | 3.33E+01 | 4.45E−05 |
| 33 | TyG | Glaucoma | rs390802 | A | G | −1.53E−02 | 1.76E−03 | 3.63E−18 | −1.77E−02 | 1.53E−02 | 2.48E−01 | 7.55E+01 | 2.94E−10 |
| 34 | TyG | Glaucoma | rs3936511 | G | A | 2.16E−02 | 1.75E−03 | 3.69E−35 | 1.40E−03 | 1.35E−02 | 9.17E−01 | 1.53E+02 | 4.69E−23 |
| 35 | TyG | Glaucoma | rs4715317 | T | G | 9.74E−03 | 1.44E−03 | 1.30E−11 | 1.14E−02 | 9.47E−03 | 2.29E−01 | 4.58E+01 | 2.26E−06 |
| 36 | TyG | Glaucoma | rs4804413 | T | C | 9.48E−03 | 1.39E−03 | 7.53E−12 | 5.36E−03 | 9.39E−03 | 5.68E−01 | 4.69E+01 | 2.38E−07 |
| 37 | TyG | Glaucoma | rs4846922 | T | C | 2.22E−02 | 1.46E−03 | 8.63E−52 | 2.71E−03 | 9.37E−03 | 7.72E−01 | 2.29E+02 | 3.35E−33 |
| 38 | TyG | Glaucoma | rs55697600 | G | A | 3.51E−02 | 3.60E−03 | 2.05E−22 | 4.32E−03 | 2.48E−02 | 8.61E−01 | 9.49E+01 | 1.09E−14 |
| 39 | TyG | Glaucoma | rs6090040 | A | C | 8.92E−03 | 1.39E−03 | 1.33E−10 | 2.83E−02 | 9.23E−03 | 2.15E−03 | 4.13E+01 | 8.34E−04 |
| 40 | TyG | Glaucoma | rs61737373 | A | G | −2.86E−02 | 2.93E−03 | 1.57E−22 | −3.98E−03 | 1.65E−02 | 8.09E−01 | 9.54E+01 | 1.25E−14 |
| 41 | TyG | Glaucoma | rs632057 | T | G | 1.54E−02 | 1.42E−03 | 2.94E−27 | 1.19E−02 | 9.27E−03 | 1.99E−01 | 1.17E+02 | 2.14E−15 |
| 42 | TyG | Glaucoma | rs6437249 | T | C | 8.38E−03 | 1.49E−03 | 1.95E−08 | −4.89E−03 | 1.04E−02 | 6.37E−01 | 3.15E+01 | 2.27E−05 |
| 43 | TyG | Glaucoma | rs6448429 | T | C | 1.27E−02 | 1.88E−03 | 1.49E−11 | −2.42E−02 | 1.31E−02 | 6.42E−02 | 4.55E+01 | 1.51E−05 |
| 44 | TyG | Glaucoma | rs6923241 | T | C | −1.09E−02 | 1.55E−03 | 1.67E−12 | 8.51E−03 | 9.73E−03 | 3.82E−01 | 4.98E+01 | 2.50E−07 |
| 45 | TyG | Glaucoma | rs71352934 | C | A | −1.63E−02 | 2.74E−03 | 2.50E−09 | −1.21E−02 | 1.55E−02 | 4.34E−01 | 3.55E+01 | 1.50E−05 |
| 46 | TyG | Glaucoma | rs71603401 | G | A | 1.25E−02 | 2.05E−03 | 1.15E−09 | −1.32E−02 | 1.72E−02 | 4.43E−01 | 3.71E+01 | 9.02E−06 |
| 47 | TyG | Glaucoma | rs7175132 | G | A | −8.12E−03 | 1.41E−03 | 9.44E−09 | −2.98E−02 | 9.33E−03 | 1.42E−03 | 3.30E+01 | 6.52E−03 |
| 48 | TyG | Glaucoma | rs72904790 | C | T | −1.37E−02 | 2.41E−03 | 1.20E−08 | −2.50E−02 | 1.98E−02 | 2.06E−01 | 3.25E+01 | 1.28E−04 |
| 49 | TyG | Glaucoma | rs73198299 | C | T | 1.23E−02 | 2.23E−03 | 3.68E−08 | −2.76E−02 | 1.42E−02 | 5.20E−02 | 3.03E+01 | 1.07E−03 |
| 50 | TyG | Glaucoma | rs77009508 | G | A | 2.24E−02 | 2.60E−03 | 5.86E−18 | −1.58E−02 | 1.93E−02 | 4.15E−01 | 7.46E+01 | 1.05E−10 |
| 51 | TyG | Glaucoma | rs78025076 | T | C | 2.71E−02 | 4.83E−03 | 1.96E−08 | 3.52E−03 | 4.88E−02 | 9.43E−01 | 3.15E+01 | 7.71E−06 |
| 52 | TyG | Glaucoma | rs79953491 | G | A | −2.37E−02 | 2.11E−03 | 2.49E−29 | 2.17E−02 | 1.54E−02 | 1.60E−01 | 1.26E+02 | 2.28E−16 |
| 53 | TyG | Glaucoma | rs8028620 | C | T | −8.98E−03 | 1.37E−03 | 6.53E−11 | −2.72E−02 | 9.17E−03 | 2.98E−03 | 4.27E+01 | 4.88E−04 |
| 54 | TyG | Glaucoma | rs8092347 | G | A | 8.12E−03 | 1.41E−03 | 8.69E−09 | −1.04E−02 | 9.23E−03 | 2.60E−01 | 3.31E+01 | 7.55E−05 |
| 55 | TyG | Glaucoma | rs9891030 | A | G | 9.94E−03 | 1.59E−03 | 4.54E−10 | 9.64E−04 | 1.15E−02 | 9.33E−01 | 3.89E+01 | 6.92E−07 |

EA = effect allele; OA = other allele; SE = standard error; SNP = single-nucleotide polymorphism; TyG = triglyceride-glucose index.

Supplementary Table 2. Age and sex stratified analysis of association between TyG and incident glaucoma

|  |  | Model 1 |  | Model 2 |  | Model 3 |  |
| --- | --- | --- | --- | --- | --- | --- | --- |
| Variables | Group | HR (95% CI) | *P*_FDR_ | HR (95% CI) | *P*_FDR_ | HR (95% CI) | *P*_FDR_ |
| TyG | Age<55 & Male | 1.12 (1.04–1.20) | 0.004 | 1.06 (0.98–1.14) | 0.146 | 1.10 (1.01–1.21) | 0.034 |
|  | Age<55 & Female | 1.18 (1.10–1.27) | <0.001 | 1.13 (1.05–1.22) | <0.001 | 1.11 (1.02–1.22) | 0.019 |
|  | Age≥55 & Male | 0.99 (0.95–1.04) | 0.776 | 0.97 (0.93–1.01) | 0.199 | 1.00 (0.95–1.05) | 0.972 |
|  | Age≥55 & Female | 1.12 (1.07–1.16) | <0.001 | 1.08 (1.03–1.12) | <0.001 | 1.09 (1.03–1.14) | 0.001 |
| TyG BMI | Age<55 & Male | 1.20 (1.11–1.30) | <0.001 | 1.10 (1.02–1.19) | 0.024 | 1.27 (1.06–1.53) | 0.013 |
|  | Age<55 & Female | 1.19 (1.13–1.27) | <0.001 | 1.15 (1.08–1.22) | <0.001 | 1.43 (1.23–1.66) | <0.001 |
|  | Age≥55 & Male | 1.09 (1.04–1.14) | <0.001 | 1.03 (0.98–1.08) | 0.203 | 1.08 (0.98–1.20) | 0.124 |
|  | Age≥55 & Female | 1.14 (1.10–1.18) | <0.001 | 1.08 (1.04–1.12) | <0.001 | 1.26 (1.15–1.37) | <0.001 |
| TyG WC | Age<55 & Male | 1.25 (1.15–1.36) | <0.001 | 1.15 (1.05–1.25) | 0.004 | 1.44 (1.21–1.71) | <0.001 |
|  | Age<55 & Female | 1.29 (1.20–1.38) | <0.001 | 1.23 (1.15–1.32) | <0.001 | 1.53 (1.32–1.77) | <0.001 |
|  | Age≥55 & Male | 1.11 (1.06–1.16) | <0.001 | 1.06 (1.01–1.11) | 0.029 | 1.19 (1.09–1.30) | <0.001 |
|  | Age≥55 & Female | 1.18 (1.14–1.23) | <0.001 | 1.12 (1.08–1.17) | <0.001 | 1.31 (1.21–1.42) | <0.001 |
| TyG WHtR | Age<55 & Male | 1.28 (1.18–1.39) | <0.001 | 1.16 (1.07–1.26) | 0.002 | 1.37 (1.18–1.60) | <0.001 |
|  | Age<55 & Female | 1.28 (1.20–1.36) | <0.001 | 1.23 (1.15–1.31) | <0.001 | 1.43 (1.28–1.61) | <0.001 |
|  | Age≥55 & Male | 1.14 (1.09–1.20) | <0.001 | 1.08 (1.03–1.13) | 0.004 | 1.17 (1.08–1.26) | <0.001 |
|  | Age≥55 & Female | 1.19 (1.15–1.24) | <0.001 | 1.13 (1.09–1.17) | <0.001 | 1.25 (1.18–1.34) | <0.001 |
| TyG WHR | Age<55 & Male | 1.26 (1.15–1.38) | <0.001 | 1.15 (1.05–1.26) | 0.006 | 1.22 (1.07–1.39) | 0.005 |
|  | Age<55 & Female | 1.35 (1.25–1.47) | <0.001 | 1.28 (1.18–1.39) | <0.001 | 1.30 (1.17–1.45) | <0.001 |
|  | Age≥55 & Male | 1.12 (1.07–1.18) | <0.001 | 1.06 (1.01–1.12) | 0.029 | 1.11 (1.04–1.18) | 0.004 |
|  | Age≥55 & Female | 1.20 (1.15–1.26) | <0.001 | 1.14 (1.09–1.20) | <0.001 | 1.17 (1.10–1.24) | <0.001 |

BMI = body mass index; CI = confidence interval; HR = hazard ratio; PFDR = false discovery rate-adjusted P value; TyG = triglyceride-glucose index; TyG BMI = triglyceride-glucose-body mass index; TyG WC = triglyceride-glucose-waist circumference; TyG WHR = triglyceride-glucose-waist-to-hip ratio; TyG WHtR = triglyceride-glucose-waist-to-height ratio.

Supplementary Table 3. Sensitivity analysis of association between TyG and incident glaucoma

| Variables | Excluded | HR (95% CI) | *P*_FDR_ | Additional adjustment | HR (95% CI) | *P*_FDR_ |
| --- | --- | --- | --- | --- | --- | --- |
| TyG | None | 1.05 (1.02–1.08) | 0.005 | None | 1.05 (1.02–1.08) | 0.003 |
|  | Cardiovascular disease | 1.05 (1.01–1.08) | 0.011 | Cardiovascular disease | 1.04 (1.01–1.08) | 0.007 |
|  | Diabetes mellitus | 0.97 (0.94–1.01) | 0.154 | Diabetes mellitus | 0.99 (0.96–1.02) | 0.541 |
|  | Hypertension | 1.01 (0.97–1.06) | 0.565 | Hypertension | 1.02 (0.99–1.05) | 0.166 |
|  | Use of insulin | 1.05 (1.02–1.08) | 0.006 | Use of insulin | 1.04 (1.01–1.08) | 0.007 |
|  | Use of anti–hypertensive drugs | 1.05 (1.02–1.09) | 0.005 | Use of anti–hypertensive drugs | 1.05 (1.02–1.08) | 0.003 |
|  | Use of cholesterol–lowering drugs | 1.06 (1.02–1.09) | 0.005 | Use of cholesterol–lowering drugs | 1.05 (1.02–1.09) | 0.003 |
| TyG BMI | None | 1.20 (1.14–1.27) | <0.001 | None | 1.20 (1.14–1.27) | <0.001 |
|  | Cardiovascular disease | 1.18 (1.11–1.26) | <0.001 | Cardiovascular disease | 1.18 (1.12–1.25) | <0.001 |
|  | Diabetes mellitus | 1.04 (0.97–1.11) | 0.225 | Diabetes mellitus | 1.08 (1.02–1.14) | 0.012 |
|  | Hypertension | 1.11 (1.01–1.21) | 0.036 | Hypertension | 1.12 (1.05–1.18) | <0.001 |
|  | Use of insulin | 1.20 (1.13–1.27) | <0.001 | Use of insulin | 1.19 (1.13–1.26) | <0.001 |
|  | Use of anti–hypertensive drugs | 1.21 (1.14–1.29) | <0.001 | Use of anti–hypertensive drugs | 1.20 (1.13–1.27) | <0.001 |
|  | Use of cholesterol–lowering drugs | 1.22 (1.14–1.29) | <0.001 | Use of cholesterol–lowering drugs | 1.21 (1.14–1.27) | <0.001 |
| TyG WC | None | 1.30 (1.23–1.37) | <0.001 | None | 1.30 (1.23–1.37) | <0.001 |
|  | Cardiovascular disease | 1.28 (1.21–1.36) | <0.001 | Cardiovascular disease | 1.28 (1.21–1.34) | <0.001 |
|  | Diabetes mellitus | 1.13 (1.07–1.20) | <0.001 | Diabetes mellitus | 1.17 (1.11–1.23) | <0.001 |
|  | Hypertension | 1.19 (1.09–1.29) | <0.001 | Hypertension | 1.21 (1.15–1.27) | <0.001 |
|  | Use of insulin | 1.29 (1.22–1.36) | <0.001 | Use of insulin | 1.28 (1.22–1.35) | <0.001 |
|  | Use of anti-hypertensive drugs | 1.31 (1.24–1.39) | <0.001 | Use of anti-hypertensive drugs | 1.30 (1.23–1.36) | <0.001 |
|  | Use of cholesterol-lowering drugs | 1.33 (1.25–1.40) | <0.001 | Use of cholesterol-lowering drugs | 1.30 (1.24–1.37) | <0.001 |
| TyG WHtR | None | 1.26 (1.20–1.31) | <0.001 | None | 1.26 (1.20–1.31) | <0.001 |
|  | Cardiovascular disease | 1.23 (1.17–1.30) | <0.001 | Cardiovascular disease | 1.24 (1.18–1.29) | <0.001 |
|  | Diabetes mellitus | 1.12 (1.07–1.18) | <0.001 | Diabetes mellitus | 1.15 (1.10–1.20) | <0.001 |
|  | Hypertension | 1.16 (1.08–1.24) | <0.001 | Hypertension | 1.17 (1.12–1.23) | <0.001 |
|  | Use of insulin | 1.25 (1.19–1.30) | <0.001 | Use of insulin | 1.24 (1.19–1.30) | <0.001 |
|  | Use of anti-hypertensive drugs | 1.26 (1.20–1.32) | <0.001 | Use of anti-hypertensive drugs | 1.25 (1.20–1.31) | <0.001 |
|  | Use of cholesterol-lowering drugs | 1.27 (1.22–1.33) | <0.001 | Use of cholesterol-lowering drugs | 1.26 (1.21–1.31) | <0.001 |
| TyG WHR | None | 1.16 (1.12–1.21) | <0.001 | None | 1.16 (1.12–1.21) | <0.001 |
|  | Cardiovascular disease | 1.15 (1.10–1.20) | <0.001 | Cardiovascular disease | 1.15 (1.10–1.19) | <0.001 |
|  | Diabetes mellitus | 1.06 (1.02–1.11) | 0.007 | Diabetes mellitus | 1.08 (1.04–1.12) | <0.001 |
|  | Hypertension | 1.09 (1.02–1.15) | 0.008 | Hypertension | 1.10 (1.06–1.15) | <0.001 |
|  | Use of insulin | 1.16 (1.11–1.20) | <0.001 | Use of insulin | 1.15 (1.11–1.20) | <0.001 |
|  | Use of anti-hypertensive drugs | 1.16 (1.11–1.21) | <0.001 | Use of anti-hypertensive drugs | 1.16 (1.12–1.21) | <0.001 |
|  | Use of cholesterol-lowering drugs | 1.18 (1.13–1.23) | <0.001 | Use of cholesterol-lowering drugs | 1.17 (1.12–1.21) | <0.001 |

BMI = body mass index; CI = confidence interval; HR = hazard ratio; PFDR = false discovery rate-adjusted P value; TyG = triglyceride-glucose index; TyG BMI = triglyceride-glucose-body mass index; TyG WC = triglyceride-glucose-waist circumference; TyG WHR = triglyceride-glucose-waist-to-hip ratio; TyG WHtR = triglyceride-glucose-waist-to-height ratio.

Supplementary Table 4. Association analysis of high/low levels of TyG and incident glaucoma

| Variables |  | Model 1 |  | Model 2 |  | Model 3 |  |
| --- | --- | --- | --- | --- | --- | --- | --- |
|  |  | HR (95% CI) | *P*_FDR_ | HR (95% CI) | *P*_FDR_ | HR (95% CI) | *P*_FDR_ |
| TyG | Normal | ref |  | ref |  | ref |  |
|  | Low | 1.02 (0.95–1.09) | 0.597 | 1.04 (0.97–1.11) | 0.288 | 1.04 (0.97–1.12) | 0.263 |
|  | High | 1.10 (1.04–1.17) | 0.003 | 1.06 (1.00–1.13) | 0.077 | 1.09 (1.02–1.16) | 0.019 |
| TyG BMI | Low | ref |  | ref |  | ref |  |
|  | High | 1.14 (1.08–1.19) | <0.001 | 1.05 (1.00–1.11) | 0.05 | 1.04 (0.97–1.12) | 0.263 |
| TyG WC | Low | ref |  | ref |  | ref |  |
|  | High | 1.21 (1.15–1.28) | <0.001 | 1.13 (1.08–1.20) | <0.001 | 1.14 (1.06–1.23) | <0.001 |
| TyG WHR | Low | ref |  | ref |  | ref |  |
|  | High | 1.25 (1.18–1.32) | <0.001 | 1.16 (1.10–1.23) | <0.001 | 1.18 (1.10–1.25) | <0.001 |
| TyG WHtR | Low | ref |  | ref |  | ref |  |
|  | High | 1.22 (1.16–1.28) | <0.001 | 1.13 (1.08–1.19) | <0.001 | 1.15 (1.07–1.22) | <0.001 |

Cut-off values of TyG and TyG related index were obtained from RCS regression model.

BMI = body mass index; CI = confidence interval; HR = hazard ratio; PFDR = false discovery rate-adjusted P value; TyG = triglyceride-glucose index; TyG BMI = triglyceride-glucose-body mass index; TyG WC = triglyceride-glucose-waist circumference; TyG WHR = triglyceride-glucose-waist-to-hip ratio; TyG WHtR = triglyceride-glucose-waist-to-height ratio.

Supplementary Table 5. Linear association analysis of IOPcc and TyG

| Variables |  | Model 1 |  |  |  | Model 2 |  |  |  | Model 3 |  |  |  |
| --- | --- | --- | --- | --- | --- | --- | --- | --- | --- | --- | --- | --- | --- |
| Y | X | Beta | SE | R^2^ | *P*_FDR_ | Beta | SE | R^2^ | *P*_FDR_ | Beta | SE | R^2^ | *P*_FDR_ |
| IOPcc | TyG | 0.049 | 0.014 | 0.024 | <0.001 | 0.065 | 0.014 | 0.026 | <0.001 | 0.055 | 0.013 | 0.128 | <0.001 |
| IOPcc | TyG BMI | 0.066 | 0.013 | 0.024 | <0.001 | 0.093 | 0.013 | 0.027 | <0.001 | 0.095 | 0.013 | 0.129 | <0.001 |
| IOPcc | TyG WC | 0.064 | 0.014 | 0.024 | <0.001 | 0.092 | 0.015 | 0.027 | <0.001 | 0.088 | 0.014 | 0.128 | <0.001 |
| IOPcc | TyG WHtR | 0.050 | 0.013 | 0.024 | <0.001 | 0.078 | 0.014 | 0.027 | <0.001 | 0.077 | 0.013 | 0.128 | <0.001 |
| IOPcc | TyG WHR | 0.037 | 0.016 | 0.024 | 0.018 | 0.063 | 0.016 | 0.026 | <0.001 | 0.053 | 0.015 | 0.128 | <0.001 |

BMI = body mass index; IOPcc = corneal-compensated intraocular pressure; PFDR = false discovery rate-adjusted P value; R2 = coefficient of determination; SE = standard error; TyG = triglyceride-glucose index; TyG BMI = triglyceride-glucose-body mass index; TyG WC = triglyceride-glucose-waist circumference; TyG WHR = triglyceride-glucose-waist-to-hip ratio; TyG WHtR = triglyceride-glucose-waist-to-height ratio.

Supplementary Table 6. Linear association analysis of retinal structures and TyG indices

| Variables |  | Model 1 |  |  |  | Model 2 |  |  |  | Model 3 |  |  |  |
| --- | --- | --- | --- | --- | --- | --- | --- | --- | --- | --- | --- | --- | --- |
| Y | X | Beta | SE | R^2^ | *P*_FDR_ | Beta | SE | R^2^ | *P*_FDR_ | Beta | SE | R^2^ | *P*_FDR_ |
| ELM.ISOS | TyG | −0.076 | 0.016 | 0.008 | <0.001 | −0.06 | 0.016 | 0.011 | <0.001 | −0.06 | 0.016 | 0.011 | <0.001 |
|  | TyG BMI | −0.106 | 0.015 | 0.008 | <0.001 | −0.088 | 0.015 | 0.011 | <0.001 | −0.089 | 0.015 | 0.012 | <0.001 |
|  | TyG WC | −0.107 | 0.016 | 0.008 | <0.001 | −0.084 | 0.017 | 0.011 | <0.001 | −0.083 | 0.017 | 0.011 | <0.001 |
|  | TyG WHtR | −0.111 | 0.015 | 0.008 | <0.001 | −0.089 | 0.016 | 0.011 | <0.001 | −0.088 | 0.016 | 0.011 | <0.001 |
|  | TyG WHR | −0.13 | 0.018 | 0.008 | <0.001 | −0.101 | 0.018 | 0.011 | <0.001 | −0.099 | 0.018 | 0.011 | <0.001 |
| INL.ELM | TyG | −0.22 | 0.048 | 0.007 | <0.001 | −0.195 | 0.048 | 0.008 | <0.001 | −0.199 | 0.049 | 0.008 | <0.001 |
|  | TyG BMI | −0.163 | 0.045 | 0.006 | <0.001 | −0.113 | 0.046 | 0.008 | 0.014 | −0.125 | 0.047 | 0.008 | 0.008 |
|  | TyG WC | −0.251 | 0.05 | 0.007 | <0.001 | −0.206 | 0.051 | 0.008 | <0.001 | −0.214 | 0.052 | 0.008 | <0.001 |
|  | TyG WHtR | −0.236 | 0.046 | 0.007 | <0.001 | −0.183 | 0.048 | 0.008 | <0.001 | −0.191 | 0.049 | 0.008 | <0.001 |
|  | TyG WHR | −0.312 | 0.055 | 0.007 | <0.001 | −0.268 | 0.056 | 0.008 | <0.001 | −0.273 | 0.057 | 0.008 | <0.001 |
| INL.RPE | TyG | −0.464 | 0.072 | 0.006 | <0.001 | −0.381 | 0.073 | 0.009 | <0.001 | −0.388 | 0.074 | 0.009 | <0.001 |
|  | TyG BMI | −0.571 | 0.068 | 0.007 | <0.001 | −0.448 | 0.07 | 0.01 | <0.001 | −0.46 | 0.071 | 0.01 | <0.001 |
|  | TyG WC | −0.665 | 0.075 | 0.007 | <0.001 | −0.544 | 0.077 | 0.01 | <0.001 | −0.55 | 0.078 | 0.01 | <0.001 |
|  | TyG WHtR | −0.669 | 0.07 | 0.007 | <0.001 | −0.536 | 0.073 | 0.01 | <0.001 | −0.543 | 0.074 | 0.01 | <0.001 |
|  | TyG WHR | −0.749 | 0.083 | 0.007 | <0.001 | −0.627 | 0.085 | 0.01 | <0.001 | −0.627 | 0.086 | 0.01 | <0.001 |
| ISOS.RPE | TyG | −0.168 | 0.029 | 0.003 | <0.001 | −0.127 | 0.03 | 0.007 | <0.001 | −0.128 | 0.03 | 0.007 | <0.001 |
|  | TyG BMI | −0.302 | 0.028 | 0.005 | <0.001 | −0.247 | 0.028 | 0.008 | <0.001 | −0.247 | 0.029 | 0.008 | <0.001 |
|  | TyG WC | −0.307 | 0.03 | 0.005 | <0.001 | −0.255 | 0.031 | 0.008 | <0.001 | −0.253 | 0.032 | 0.008 | <0.001 |
|  | TyG WHtR | −0.322 | 0.028 | 0.005 | <0.001 | −0.265 | 0.029 | 0.008 | <0.001 | −0.264 | 0.03 | 0.008 | <0.001 |
|  | TyG WHR | −0.307 | 0.034 | 0.004 | <0.001 | −0.257 | 0.034 | 0.008 | <0.001 | −0.255 | 0.035 | 0.008 | <0.001 |
| IPL | TyG | −0.091 | 0.042 | 0.013 | 0.03 | −0.105 | 0.043 | 0.014 | 0.015 | −0.119 | 0.043 | 0.015 | 0.007 |
|  | TyG BMI | −0.099 | 0.04 | 0.013 | 0.015 | −0.119 | 0.041 | 0.014 | 0.005 | −0.136 | 0.041 | 0.015 | 0.001 |
|  | TyG WC | −0.172 | 0.044 | 0.013 | <0.001 | −0.186 | 0.045 | 0.014 | <0.001 | −0.211 | 0.045 | 0.015 | <0.001 |
|  | TyG WHtR | −0.168 | 0.041 | 0.013 | <0.001 | −0.185 | 0.042 | 0.014 | <0.001 | −0.206 | 0.043 | 0.015 | <0.001 |
|  | TyG WHR | −0.183 | 0.048 | 0.013 | <0.001 | −0.195 | 0.049 | 0.014 | <0.001 | −0.219 | 0.05 | 0.015 | <0.001 |
| INL | TyG | −0.003 | 0.019 | 0.006 | 0.934 | −0.019 | 0.019 | 0.007 | 0.432 | −0.018 | 0.02 | 0.007 | 0.438 |
|  | TyG BMI | 0.071 | 0.018 | 0.006 | 0.001 | 0.048 | 0.019 | 0.007 | 0.032 | 0.048 | 0.019 | 0.007 | 0.032 |
|  | TyG WC | 0.054 | 0.02 | 0.006 | 0.031 | 0.031 | 0.02 | 0.007 | 0.219 | 0.031 | 0.021 | 0.007 | 0.219 |
|  | TyG WHtR | 0.056 | 0.019 | 0.006 | 0.021 | 0.03 | 0.019 | 0.007 | 0.219 | 0.03 | 0.019 | 0.007 | 0.219 |
|  | TyG WHR | 0.028 | 0.022 | 0.006 | 0.306 | 0.002 | 0.022 | 0.007 | 0.942 | 0.004 | 0.023 | 0.007 | 0.934 |
| RNFL | TyG | −0.137 | 0.034 | 0.007 | <0.001 | −0.105 | 0.035 | 0.009 | 0.002 | −0.12 | 0.035 | 0.009 | <0.001 |
|  | TyG BMI | −0.213 | 0.032 | 0.008 | <0.001 | −0.167 | 0.033 | 0.009 | <0.001 | −0.182 | 0.034 | 0.01 | <0.001 |
|  | TyG WC | −0.189 | 0.035 | 0.008 | <0.001 | −0.138 | 0.036 | 0.009 | <0.001 | −0.159 | 0.037 | 0.009 | <0.001 |
|  | TyG WHtR | −0.25 | 0.033 | 0.008 | <0.001 | −0.198 | 0.034 | 0.01 | <0.001 | −0.215 | 0.035 | 0.01 | <0.001 |
|  | TyG WHR | −0.21 | 0.039 | 0.008 | <0.001 | −0.155 | 0.04 | 0.009 | <0.001 | −0.174 | 0.041 | 0.009 | <0.001 |
| VDD | TyG | −0.097 | 0.078 | 0.002 | 0.457 | −0.18 | 0.079 | 0.005 | 0.169 | −0.181 | 0.079 | 0.005 | 0.169 |
|  | TyG BMI | 0.075 | 0.074 | 0.002 | 0.465 | −0.042 | 0.076 | 0.004 | 0.735 | −0.041 | 0.076 | 0.004 | 0.735 |
|  | TyG WC | 0.038 | 0.081 | 0.002 | 0.738 | −0.091 | 0.083 | 0.004 | 0.464 | −0.09 | 0.083 | 0.005 | 0.464 |
|  | TyG WHtR | 0.026 | 0.076 | 0.002 | 0.785 | −0.11 | 0.078 | 0.004 | 0.406 | −0.109 | 0.078 | 0.005 | 0.406 |
|  | TyG WHR | 0.001 | 0.09 | 0.002 | 0.994 | −0.146 | 0.092 | 0.004 | 0.406 | −0.147 | 0.092 | 0.005 | 0.406 |
| RPE | TyG | 0.121 | 0.126 | <0.001 | 0.632 | 0.024 | 0.128 | 0.002 | 0.953 | 0.04 | 0.13 | 0.002 | 0.946 |
|  | TyG BMI | 0.003 | 0.119 | <0.001 | 0.978 | −0.158 | 0.123 | 0.002 | 0.594 | −0.127 | 0.124 | 0.002 | 0.632 |
|  | TyG WC | −0.126 | 0.131 | <0.001 | 0.632 | −0.273 | 0.135 | 0.002 | 0.45 | −0.257 | 0.137 | 0.002 | 0.45 |
|  | TyG WHtR | −0.017 | 0.123 | <0.001 | 0.953 | −0.183 | 0.127 | 0.002 | 0.594 | −0.165 | 0.128 | 0.002 | 0.594 |
|  | TyG WHR | 0.085 | 0.144 | <0.001 | 0.925 | −0.059 | 0.148 | 0.002 | 0.946 | −0.054 | 0.15 | 0.002 | 0.946 |
| Macular | TyG | −0.695 | 0.113 | 0.009 | <0.001 | −0.611 | 0.115 | 0.011 | <0.001 | −0.645 | 0.117 | 0.011 | <0.001 |
|  | TyG BMI | −0.812 | 0.107 | 0.009 | <0.001 | −0.686 | 0.11 | 0.011 | <0.001 | −0.73 | 0.112 | 0.011 | <0.001 |
|  | TyG WC | −0.971 | 0.118 | 0.009 | <0.001 | −0.837 | 0.121 | 0.011 | <0.001 | −0.889 | 0.123 | 0.012 | <0.001 |
|  | TyG WHtR | −1.03 | 0.11 | 0.01 | <0.001 | −0.889 | 0.114 | 0.012 | <0.001 | −0.933 | 0.115 | 0.012 | <0.001 |
|  | TyG WHR | −1.114 | 0.13 | 0.009 | <0.001 | −0.975 | 0.133 | 0.012 | <0.001 | −1.016 | 0.135 | 0.012 | <0.001 |
| Macular.V | TyG | −0.016 | 0.003 | 0.024 | <0.001 | −0.016 | 0.003 | 0.026 | <0.001 | −0.016 | 0.003 | 0.026 | <0.001 |
|  | TyG BMI | −0.019 | 0.002 | 0.024 | <0.001 | −0.019 | 0.003 | 0.027 | <0.001 | −0.019 | 0.003 | 0.027 | <0.001 |
|  | TyG WC | −0.026 | 0.003 | 0.026 | <0.001 | −0.025 | 0.003 | 0.028 | <0.001 | −0.025 | 0.003 | 0.028 | <0.001 |
|  | TyG WHtR | −0.027 | 0.003 | 0.026 | <0.001 | −0.025 | 0.003 | 0.029 | <0.001 | −0.025 | 0.003 | 0.029 | <0.001 |
|  | TyG WHR | −0.029 | 0.003 | 0.026 | <0.001 | −0.027 | 0.003 | 0.028 | <0.001 | −0.027 | 0.003 | 0.028 | <0.001 |
| CD | TyG | −0.002 | 0.001 | 0.001 | 0.026 | −0.002 | 0.001 | 0.002 | 0.067 | −0.002 | 0.001 | 0.052 | 0.047 |
|  | TyG BMI | −0.006 | 0.001 | 0.002 | <0.001 | −0.006 | 0.001 | 0.003 | <0.001 | −0.005 | 0.001 | 0.053 | <0.001 |
|  | TyG WC | −0.005 | 0.001 | 0.002 | <0.001 | −0.004 | 0.001 | 0.002 | <0.001 | −0.004 | 0.001 | 0.052 | <0.001 |
|  | TyG WHtR | −0.005 | 0.001 | 0.002 | <0.001 | −0.005 | 0.001 | 0.003 | <0.001 | −0.004 | 0.001 | 0.052 | <0.001 |
|  | TyG WHR | −0.004 | 0.001 | 0.001 | <0.001 | −0.003 | 0.001 | 0.002 | 0.003 | −0.003 | 0.001 | 0.052 | 0.001 |

Beta = regression coefficient; CD = cup-to-disc ratio; ELM = external limiting membrane; INL = inner nuclear layer; IOP = intraocular pressure; IPL = inner plexiform layer; ISOS = inner and outer photoreceptor segments; Macular.V = macular volume; PFDR = false discovery rate-adjusted P value; RNFL = retinal nerve fiber layer; RPE = retinal pigment epithelium; R2 = coefficient of determination; SE = standard error; TyG = triglyceride-glucose index; TyG BMI = triglyceride-glucose-body mass index; TyG WC = triglyceride-glucose-waist circumference; TyG WHR = triglyceride-glucose-waist-to-hip ratio; TyG WHtR = triglyceride-glucose-waist-to-height ratio; VDD = vertical disc diameter; X = exposure; Y = outcome.

Supplementary Table 7. Spearman correlation analysis of plasma biochemical markers and TyG in glaucoma

| Biomarker |  | Spearman R | *P*_FDR_ | Biomarker |  | Spearman R | *P*_FDR_ |
| --- | --- | --- | --- | --- | --- | --- | --- |
| Alanine.aminotransferase | TyG | 0.297 | <0.001 | Phosphate | TyG | −0.056 | <0.001 |
|  | TyG_BMI | 0.380 | <0.001 |  | TyG_BMI | −0.101 | <0.001 |
|  | TyG_WC | 0.422 | <0.001 |  | TyG_WC | −0.170 | <0.001 |
|  | TyG_WHtR | 0.396 | <0.001 |  | TyG_WHtR | −0.110 | <0.001 |
|  | TyG_WHR | 0.412 | <0.001 |  | TyG_WHR | −0.180 | <0.001 |
| Albumin | TyG | 0.045 | <0.001 | SHBG | TyG | −0.397 | <0.001 |
|  | TyG_BMI | −0.055 | <0.001 |  | TyG_BMI | −0.488 | <0.001 |
|  | TyG_WC | −0.033 | 0.008 |  | TyG_WC | −0.559 | <0.001 |
|  | TyG_WHtR | −0.045 | <0.001 |  | TyG_WHtR | −0.512 | <0.001 |
|  | TyG_WHR | 0.022 | 0.071 |  | TyG_WHR | −0.540 | <0.001 |
| Alkaline.phosphatase | TyG | 0.150 | <0.001 | Testosterone | TyG | 0.001 | 0.937 |
|  | TyG_BMI | 0.176 | <0.001 |  | TyG_BMI | −0.002 | 0.913 |
|  | TyG_WC | 0.130 | <0.001 |  | TyG_WC | 0.261 | <0.001 |
|  | TyG_WHtR | 0.181 | <0.001 |  | TyG_WHtR | 0.079 | <0.001 |
|  | TyG_WHR | 0.086 | <0.001 |  | TyG_WHR | 0.384 | <0.001 |
| Aspartate_aminotransferase | TyG | 0.098 | <0.001 | Total_bilirubin | TyG | −0.146 | <0.001 |
|  | TyG_BMI | 0.123 | <0.001 |  | TyG_BMI | −0.098 | <0.001 |
|  | TyG_WC | 0.156 | <0.001 |  | TyG_WC | −0.003 | 0.867 |
|  | TyG_WHtR | 0.133 | <0.001 |  | TyG_WHtR | −0.094 | <0.001 |
|  | TyG_WHR | 0.175 | <0.001 |  | TyG_WHR | 0.043 | <0.001 |
| Creactive_protein | TyG | 0.241 | <0.001 | Total_protein | TyG | 0.070 | <0.001 |
|  | TyG_BMI | 0.436 | <0.001 |  | TyG_BMI | 0.059 | <0.001 |
|  | TyG_WC | 0.380 | <0.001 |  | TyG_WC | 0.053 | <0.001 |
|  | TyG_WHtR | 0.432 | <0.001 |  | TyG_WHtR | 0.066 | <0.001 |
|  | TyG_WHR | 0.279 | <0.001 |  | TyG_WHR | 0.061 | <0.001 |
| Calcium | TyG | 0.103 | <0.001 | Urate | TyG | 0.280 | <0.001 |
|  | TyG_BMI | 0.025 | 0.048 |  | TyG_BMI | 0.404 | <0.001 |
|  | TyG_WC | −0.008 | 0.556 |  | TyG_WC | 0.520 | <0.001 |
|  | TyG_WHtR | 0.028 | 0.025 |  | TyG_WHtR | 0.435 | <0.001 |
|  | TyG_WHR | −0.002 | 0.892 |  | TyG_WHR | 0.511 | <0.001 |
| Creatinine | TyG | 0.090 | <0.001 | Urea | TyG | 0.101 | <0.001 |
|  | TyG_BMI | 0.135 | <0.001 |  | TyG_BMI | 0.103 | <0.001 |
|  | TyG_WC | 0.293 | <0.001 |  | TyG_WC | 0.120 | <0.001 |
|  | TyG_WHtR | 0.164 | <0.001 |  | TyG_WHtR | 0.112 | <0.001 |
|  | TyG_WHR | 0.349 | <0.001 |  | TyG_WHR | 0.119 | <0.001 |
| CystainC | TyG | 0.208 | <0.001 | VitaminD | TyG | −0.170 | <0.001 |
|  | TyG_BMI | 0.342 | <0.001 |  | TyG_BMI | −0.219 | <0.001 |
|  | TyG_WC | 0.372 | <0.001 |  | TyG_WC | −0.206 | <0.001 |
|  | TyG_WHtR | 0.359 | <0.001 |  | TyG_WHtR | −0.230 | <0.001 |
|  | TyG_WHR | 0.316 | <0.001 |  | TyG_WHR | −0.157 | <0.001 |
| Bilirubin | TyG | −0.144 | <0.001 | PUFAs | TyG | 0.305 | <0.001 |
|  | TyG_BMI | −0.056 | <0.001 |  | TyG_BMI | 0.050 | 0.002 |
|  | TyG_WC | 0.033 | 0.013 |  | TyG_WC | −0.015 | 0.385 |
|  | TyG_WHtR | −0.045 | 0.001 |  | TyG_WHtR | 0.059 | <0.001 |
|  | TyG_WHR | 0.064 | <0.001 |  | TyG_WHR | 0.001 | 0.937 |
| Gamma_glutamyltransferase | TyG | 0.307 | <0.001 | Omega3 | TyG | 0.260 | <0.001 |
|  | TyG_BMI | 0.360 | <0.001 |  | TyG_BMI | 0.064 | 0.000 |
|  | TyG_WC | 0.433 | <0.001 |  | TyG_WC | 0.021 | 0.206 |
|  | TyG_WHtR | 0.404 | <0.001 |  | TyG_WHtR | 0.074 | <0.001 |
|  | TyG_WHR | 0.440 | <0.001 |  | TyG_WHR | 0.035 | 0.034 |
| HbA1c | TyG | 0.274 | <0.001 | Omega6 | TyG | 0.280 | <0.001 |
|  | TyG_BMI | 0.301 | <0.001 |  | TyG_BMI | 0.042 | 0.010 |
|  | TyG_WC | 0.296 | <0.001 |  | TyG_WC | −0.023 | 0.167 |
|  | TyG_WHtR | 0.318 | <0.001 |  | TyG_WHtR | 0.048 | 0.003 |
|  | TyG_WHR | 0.270 | <0.001 |  | TyG_WHR | −0.009 | 0.591 |
| IGF1 | TyG | −0.008 | 0.556 | LA | TyG | 0.270 | <0.001 |
|  | TyG_BMI | −0.065 | <0.001 |  | TyG_BMI | 0.011 | 0.507 |
|  | TyG_WC | −0.018 | 0.155 |  | TyG_WC | −0.034 | 0.036 |
|  | TyG_WHtR | −0.072 | <0.001 |  | TyG_WHtR | 0.021 | 0.209 |
|  | TyG_WHR | 0.025 | 0.043 |  | TyG_WHR | −0.009 | 0.582 |
| LPa | TyG | −0.038 | 0.006 | DHA | TyG | −0.084 | <0.001 |
|  | TyG_BMI | −0.001 | 0.963 |  | TyG_BMI | −0.172 | <0.001 |
|  | TyG_WC | −0.027 | 0.054 |  | TyG_WC | −0.233 | <0.001 |
|  | TyG_WHtR | −0.018 | 0.196 |  | TyG_WHtR | −0.186 | <0.001 |
|  | TyG_WHR | −0.043 | 0.002 |  | TyG_WHR | −0.232 | <0.001 |

BMI = body mass index; DHA = docosahexaenoic acid; HbA1c = glycated hemoglobin; IGF1 = insulin-like growth factor 1; LA = linoleic acid; LPa = lipoprotein(a); PFDR = false discovery rate-adjusted P value; PUFAs = polyunsaturated fatty acids; SHBG = sex hormone-binding globulin; Spearman R = Spearman correlation coefficient; TyG = triglyceride-glucose index; TyG BMI = triglyceride-glucose-body mass index; TyG WC = triglyceride-glucose-waist circumference; TyG WHR = triglyceride-glucose-waist-to-hip ratio; TyG WHtR = triglyceride-glucose-waist-to-height ratio.

Supplementary Table 8. Performance of XGBoost machine learning prediction model for incident glaucoma risk

| Variables | C-index | AUC | Specificity | Sensitivity | Accuracy |
| --- | --- | --- | --- | --- | --- |
| TG | 0.811 (0.785–0.837) | 0.802 | 0.706 | 0.76 | 0.707 |
| Glucose | 0.805 (0.751–0.859) | 0.799 | 0.686 | 0.717 | 0.687 |
| TyG | 0.825 (0.776–0.874) | 0.798 | 0.716 | 0.722 | 0.716 |
| TyG BMI | 0.814 (0.758–0.870) | 0.806 | 0.705 | 0.72 | 0.705 |
| TyG WC | 0.831 (0.784–0.877) | 0.785 | 0.713 | 0.76 | 0.713 |
| TyG WHtR | 0.851 (0.797–0.905) | 0.823 | 0.704 | 0.788 | 0.706 |
| TyG WHR | 0.849 (0.804–0.893) | 0.821 | 0.711 | 0.76 | 0.711 |

AUC values are reported as point estimates; 95% confidence intervals are shown in parentheses for the C-index.

AUC = area under the curve; BMI = body mass index; C-index = concordance index; TG = triglycerides; TyG = triglyceride-glucose index; TyG BMI = triglyceride-glucose-body mass index; TyG WC = triglyceride-glucose-waist circumference; TyG WHR = triglyceride-glucose-waist-to-hip ratio; TyG WHtR = triglyceride-glucose-waist-to-height ratio.

Supplementary Table 9. Mendelian Randomization analysis of TyG and glaucoma risk

| Exposure | Outcome | Method | nSNP | b | SE | OR (95% CI) | *P* |
| --- | --- | --- | --- | --- | --- | --- | --- |
| TyG | Glaucoma | MR Egger | 55 | −0.06 | 0.25 | 0.94(0.58–1.54) | 0.813 |
| TyG | Glaucoma | Weighted median | 55 | 0.18 | 0.17 | 1.19(0.85–1.67) | 0.301 |
| TyG | Glaucoma | Inverse variance weighted | 55 | 0.24 | 0.12 | 1.27(1.00–1.62) | 0.046 |
| TyG | Glaucoma | Simple mode | 55 | 0.32 | 0.3 | 1.38(0.76–2.49) | 0.292 |
| TyG | Glaucoma | Weighted mode | 55 | 0.24 | 0.17 | 1.27(0.91–1.77) | 0.172 |

CI = confidence interval; MR = Mendelian randomization; nSNP = number of single-nucleotide polymorphisms; OR = odds ratio; SE = standard error; TyG = triglyceride-glucose index.

Supplementary Table 10. Mendelian randomization analysis of TyG and diabetes mellitus risk

| Exposure | Outcome | Method | nSNP | b | SE | OR (95% CI) | *P* |
| --- | --- | --- | --- | --- | --- | --- | --- |
| TyG | DM | MR Egger | 68 | −0.22 | 0.26 | 0.80(0.49–1.33) | 0.396 |
| TyG | DM | Weighted median | 68 | 0.13 | 0.17 | 1.14(0.81–1.61) | 0.442 |
| TyG | DM | Inverse variance weighted | 68 | 0.48 | 0.16 | 1.61(1.17–2.23) | 0.004 |
| TyG | DM | Simple mode | 68 | 0.57 | 0.36 | 1.76(0.87–3.58) | 0.121 |
| TyG | DM | Weighted mode | 68 | 0.08 | 0.14 | 1.09(0.83–1.42) | 0.551 |

CI = confidence interval; DM = diabetes mellitus; MR = Mendelian randomization; nSNP = number of single-nucleotide polymorphisms; OR = odds ratio; SE = standard error; TyG = triglyceride-glucose index.

Supplementary Table 11. Mediation mendelian randomization of TyG, diabetes mellitus and glaucoma

| Effect type | β | SE | *P* | Interpretation DM |
| --- | --- | --- | --- | --- |
| TyG → Glaucoma | 0.24 | 0.12 | 0.046 | Significant total effect of TyG on glaucoma |
| TyG → DM | 0.48 | 0.16 | 0.004 | Significant effect of TyG on DM |
| DM → Glaucoma | 0.07 | 0.02 | 1.80×10⁻⁴ | Significant effect of DM on glaucoma |
| Proportion mediated | 14.0% |  |  | DM mediates 14.0% of the total effect of TyG on glaucoma |

DM = diabetes mellitus; SE = standard error; TyG = triglyceride-glucose index

Supplementary Table 12. Mendelian randomization of OCT and glaucoma

| Exposure | Outcome | nSNP | b | SE | OR (95% CI) | *P* | *PFDR* |
| --- | --- | --- | --- | --- | --- | --- | --- |
| ELM_ISOS_thickness_left | Glaucoma | 18 | −0.05 | 0.11 | 0.95(0.77–1.18) | 6.68E−01 | 8.15E−01 |
| GCIPL_thickness_right | Glaucoma | 34 | 0.04 | 0.05 | 1.04(0.94–1.15) | 4.33E−01 | 7.67E−01 |
| INL_thickness_left | Glaucoma | 56 | −0.03 | 0.04 | 0.97(0.89–1.05) | 4.27E−01 | 7.67E−01 |
| INL_thickness_right | Glaucoma | 58 | −0.03 | 0.04 | 0.97(0.89–1.04) | 3.75E−01 | 7.67E−01 |
| RNFL_thickness_left | Glaucoma | 16 | −0.12 | 0.06 | 0.89(0.79–1.01) | 6.18E−02 | 3.32E−01 |
| RNFL_thickness_right | Glaucoma | 21 | −0.16 | 0.09 | 0.85(0.71–1.03) | 9.24E−02 | 3.32E−01 |
| Disc_diameter_trans_left | Glaucoma | 43 | 0.12 | 0.05 | 1.12(1.01–1.25) | 2.97E−02 | 3.32E−01 |
| ELM_ISOS_thickness_central_subfield_left | Glaucoma | 28 | −0.03 | 0.05 | 0.97(0.88–1.07) | 5.30E−01 | 8.15E−01 |
| ELM_ISOS_thickness_central_subfield_right | Glaucoma | 30 | −0.03 | 0.05 | 0.98(0.89–1.07) | 5.87E−01 | 8.15E−01 |
| ELM_ISOS_thickness_inner_subfield_left | Glaucoma | 49 | −0.02 | 0.05 | 0.98(0.89–1.07) | 6.35E−01 | 8.15E−01 |
| ELM_ISOS_thickness_inner_subfield_right | Glaucoma | 49 | −0.02 | 0.05 | 0.98(0.89–1.08) | 7.38E−01 | 8.15E−01 |
| ELM_ISOS_thickness_right | Glaucoma | 29 | 0.04 | 0.08 | 1.04(0.90–1.21) | 5.80E−01 | 8.15E−01 |
| ELM_ISOS_thickness_outer_subfield_left | Glaucoma | 18 | −0.02 | 0.12 | 0.98(0.77–1.24) | 8.45E−01 | 9.04E−01 |
| ELM_ISOS_thickness_outer_subfield_right | Glaucoma | 20 | −0.03 | 0.1 | 0.97(0.80–1.17) | 7.44E−01 | 8.15E−01 |
| INL_ELM_thickness_central_subfield_left | Glaucoma | 39 | 0 | 0.05 | 1.00(0.90–1.10) | 9.29E−01 | 9.72E−01 |
| INL_ELM_thickness_central_subfield_right | Glaucoma | 38 | 0.03 | 0.05 | 1.03(0.94–1.13) | 5.75E−01 | 8.15E−01 |
| INL_ELM_thickness_inner_subfield_left | Glaucoma | 71 | 0.05 | 0.04 | 1.06(0.98–1.14) | 1.50E−01 | 4.06E−01 |
| INL_ELM_thickness_inner_subfield_right | Glaucoma | 67 | 0.06 | 0.04 | 1.06(0.99–1.15) | 1.14E−01 | 3.32E−01 |
| INL_ELM_thickness_outer_subfield_left | Glaucoma | 74 | 0.05 | 0.03 | 1.05(1.00–1.11) | 7.02E−02 | 3.32E−01 |
| INL_ELM_thickness_outer_subfield_right | Glaucoma | 72 | 0.04 | 0.03 | 1.04(0.99–1.10) | 1.11E−01 | 3.32E−01 |
| INL_RPE_thickness_central_subfield_left | Glaucoma | 21 | 0.02 | 0.06 | 1.02(0.91–1.16) | 6.97E−01 | 8.15E−01 |
| INL_RPE_thickness_central_subfield_right | Glaucoma | 32 | −0.02 | 0.06 | 0.98(0.87–1.10) | 7.12E−01 | 8.15E−01 |
| INL_ELM_thickness_left | Glaucoma | 75 | 0.05 | 0.03 | 1.05(0.99–1.11) | 1.08E−01 | 3.32E−01 |
| INL_RPE_thickness_inner_subfield_left | Glaucoma | 64 | 0.05 | 0.04 | 1.05(0.98–1.13) | 1.91E−01 | 4.61E−01 |
| INL_RPE_thickness_inner_subfield_right | Glaucoma | 64 | 0.06 | 0.03 | 1.06(0.99–1.13) | 7.73E−02 | 3.32E−01 |
| INL_RPE_thickness_outer_subfield_left | Glaucoma | 86 | 0.06 | 0.03 | 1.06(1.00–1.12) | 4.37E−02 | 3.32E−01 |
| INL_RPE_thickness_outer_subfield_right | Glaucoma | 79 | 0.05 | 0.03 | 1.05(0.99–1.11) | 1.15E−01 | 3.32E−01 |
| ISOS_RPE_thickness_central_subfield_left | Glaucoma | 42 | −0.07 | 0.05 | 0.94(0.85–1.03) | 1.84E−01 | 4.61E−01 |
| ISOS_RPE_thickness_central_subfield_right | Glaucoma | 39 | −0.05 | 0.05 | 0.95(0.85–1.05) | 3.06E−01 | 6.71E−01 |
| ISOS_RPE_thickness_inner_subfield_left | Glaucoma | 43 | −0.04 | 0.05 | 0.96(0.88–1.06) | 4.10E−01 | 7.67E−01 |
| ISOS_RPE_thickness_inner_subfield_right | Glaucoma | 44 | −0.02 | 0.05 | 0.98(0.90–1.07) | 6.63E−01 | 8.15E−01 |
| ISOS_RPE_thickness_outer_subfield_left | Glaucoma | 46 | −0.02 | 0.04 | 0.98(0.90–1.06) | 5.98E−01 | 8.15E−01 |
| ISOS_RPE_thickness_outer_subfield_right | Glaucoma | 46 | −0.02 | 0.05 | 0.98(0.90–1.08) | 7.37E−01 | 8.15E−01 |
| INL_ELM_thickness_right | Glaucoma | 76 | 0.05 | 0.03 | 1.05(0.99–1.11) | 8.75E−02 | 3.32E−01 |
| mean_of_vertical_disc_diameter_left | Glaucoma | 32 | 0.15 | 0.07 | 1.16(1.01–1.32) | 2.96E−02 | 3.32E−01 |
| overall_average_retinal_pigment_epithelium_thickness_left | Glaucoma | 28 | 0.05 | 0.07 | 1.05(0.92–1.20) | 4.80E−01 | 8.15E−01 |
| overall_average_retinal_pigment_epithelium_thickness_right | Glaucoma | 27 | 0 | 0.05 | 1.00(0.90–1.11) | 9.81E−01 | 9.81E−01 |
| overall_macular_thickness_left | Glaucoma | 58 | 0.02 | 0.05 | 1.02(0.92–1.13) | 6.95E−01 | 8.15E−01 |
| overall_macular_thickness_right | Glaucoma | 64 | 0.02 | 0.05 | 1.02(0.93–1.13) | 6.67E−01 | 8.15E−01 |
| VCDR_left | Glaucoma | 30 | 0.42 | 0.09 | 1.52(1.28–1.80) | 1.25E−06 | 2.87E−05 |
| VCDR_regressed_left | Glaucoma | 33 | 0.42 | 0.08 | 1.52(1.29–1.79) | 5.96E−07 | 2.74E−05 |
| INL_RPE_thickness_left | Glaucoma | 86 | 0.06 | 0.03 | 1.06(1.00–1.12) | 4.97E−02 | 3.32E−01 |
| INL_RPE_thickness_right | Glaucoma | 89 | 0.05 | 0.03 | 1.06(1.00–1.12) | 5.87E−02 | 3.32E−01 |
| ISOS_RPE_thickness_left | Glaucoma | 48 | −0.05 | 0.04 | 0.96(0.88–1.03) | 2.48E−01 | 5.71E−01 |
| ISOS_RPE_thickness_right | Glaucoma | 48 | 0 | 0.05 | 1.00(0.91–1.09) | 9.71E−01 | 9.81E−01 |
| GCIPL_thickness_left | Glaucoma | 31 | 0.04 | 0.05 | 1.04(0.95-1.13) | 4.34E−01 | 7.67E−01 |

CI = confidence interval; ELM = external limiting membrane; GCIPL = ganglion cell-inner plexiform layer; INL = inner nuclear layer; ISOS = inner and outer photoreceptor segments; nSNP = number of single-nucleotide polymorphisms; OCT = optical coherence tomography; OR = odds ratio; PFDR = false discovery rate-adjusted P value; RNFL = retinal nerve fiber layer; RPE = retinal pigment epithelium; SE = standard error; VCDR = vertical cup-to-disc ratio.

Supplementary Table 13. Mediation Mendelian randomization of TyG, vertical cup-to-disc ratio and glaucoma

| Effect type | β | SE | *P* | Interpretation |
| --- | --- | --- | --- | --- |
| TyG → Glaucoma | 0.24 | 0.12 | 0.046 | Significant total effect of TyG on glaucoma |
| TyG → VCDR | 0.16 | 0.07 | 0.032 | Significant effect of TyG on VCDR |
| VCDR → Glaucoma | 0.42 | 0.08 | 5.96×10⁻⁷ | Significant effect of VCDR on glaucoma |
| Proportion mediated | 28.0% |  |  | VCDR mediates 28.0% of the total effect of TyG on glaucoma |

SE= standard error; TyG = triglyceride-glucose index; VCDR = vertical cup-to-disc ratio

Supplementary Table 14. Mendelian randomization analysis of TyG and IOP risk

| Exposure | Outcome | Method | nSNP | b | SE | OR (95% CI) | *P* |
| --- | --- | --- | --- | --- | --- | --- | --- |
| TyG | IOP | MR Egger | 66 | −0.09 | 0.09 | 0.92(0.76–1.10) | 0.358 |
| TyG | IOP | Weighted median | 66 | −0.05 | 0.08 | 0.95(0.82–1.11) | 0.544 |
| TyG | IOP | Inverse variance weighted | 66 | 0.05 | 0.06 | 1.05(0.94–1.18) | 0.376 |
| TyG | IOP | Simple mode | 66 | −0.21 | 0.16 | 0.81(0.59–1.11) | 0.198 |
| TyG | IOP | Weighted mode | 66 | −0.07 | 0.07 | 0.93(0.82–1.07) | 0.338 |

CI = confidence interval; IOP = intraocular pressure; nSNP = number of single-nucleotide polymorphisms; OR = odds ratio; SE = standard error; TyG = triglyceride-glucose index.

Supplementary Figure 1. Mendelian randomization (MR) of forest plot with triglyceride-glucose (TyG) and glaucoma

Supplementary Figure 2. Mendelian randomization (MR) of scatter plot with triglyceride-glucose (TyG) and glaucoma. SNP, single-nucleotide polymorphism
